# Mapping gray and white matter volume abnormalities in early-onset psychosis: an ENIGMA multicenter voxel-based morphometry study

**DOI:** 10.1038/s41380-023-02343-1

**Published:** 2024-01-10

**Authors:** Shuqing Si, Anbreen Bi, Zhaoying Yu, Cheryl See, Sinead Kelly, Sonia Ambrogi, Celso Arango, Inmaculada Baeza, Nerisa Banaj, Michael Berk, Josefina Castro-Fornieles, Benedicto Crespo-Facorro, Jacob J. Crouse, Covadonga M. Díaz-Caneja, Anne-Kathrin Fett, Adriana Fortea, Sophia Frangou, Benjamin I. Goldstein, Ian B. Hickie, Joost Janssen, Kody G. Kennedy, Lydia Krabbendam, Marinos Kyriakopoulos, Bradley J. MacIntosh, Pedro Morgado, Stener Nerland, Saül Pascual-Diaz, Maria Picó-Pérez, Fabrizio Piras, Bjørn Rishovd Rund, Elena de la Serna, Gianfranco Spalletta, Gisela Sugranyes, Chao Suo, Diana Tordesillas-Gutiérrez, Daniela Vecchio, Joaquim Radua, Philip McGuire, Sophia I. Thomopoulos, Neda Jahanshad, Paul M. Thompson, Claudia Barth, Ingrid Agartz, Anthony James, Matthew J. Kempton

**Affiliations:** 1 Department of Psychosis Studies, Institute of Psychiatry, Psychology & Neuroscience, King’s College London, London, United Kingdom; 2 Laboratory of Neuropsychiatry, Santa Lucia Foundation IRCCS, Rome, Italy; 3 Department of Child and Adolescent Psychiatry, Institute of Psychiatry and Mental Health, Hospital General Universitario Gregorio Marañón, IiSGM, CIBERSAM, Madrid, Spain; 4 School of Medicine, Universidad Complutense, Madrid, Spain; 5 Department of Child and Adolescent Psychiatry and Psychology, 2021SGR01319, Hospital Clinic Barcelona. CIBERSAM-ISCIII. Fundació de Recerca Clínic Barcelona - August Pi i Sunyer Biomedical Research Institute (FCRB-IDIBAPS). Institute of Neuroscience, Department of Medicine, University of Barcelona, Barcelona, Spain; 6 Deakin University, Institute for Mental and Physical Health and Clinical Translation, School of Medicine, Barwon Health, Geelong, Australia; 7 Hospital Universitario Virgen del Rocío, Universidad de Sevilla, Department of Psychiatry, CIBERSAM, IBiS-CSIC, Sevilla, Spain; 8 Brain and Mind Centre, University of Sydney, Sydney, Australia; 9 Department of Psychology, City, University of London, London, UK; 10 Department of Psychiatry, University of British Columbia, Vancouver, BC Canada; 11 Icahn School of Medicine at Mount Sinai, New York, NY USA; 12 Centre for Youth Bipolar Disorder, Centre for Addiction and Mental Health, Toronto, ON Canada; 13 Department of Pharmacology & Toxicology, University of Toronto, Toronto, ON Canada; 14 Department of Psychiatry, University of Toronto, Toronto, Canada; 15 Department of Clinical, Neuro and Developmental Psychology, Faculty of Behavioural and Movement Sciences, Institute for Brain and Behaviour (IBBA) Amsterdam, Vrije Universiteit Amsterdam, Amsterdam, The Netherlands; 16 1st Department of Psychiatry, National and Kapodistrian University of Athens, Athens, Greece; 17 Department of Child and Adolescent Psychiatry, Institute of Psychiatry, Psychology & Neuroscience, King’s College London, London, United Kingdom; 18 South London and Maudsley NHS Foundation Trust, London, United Kingdom; 19 Department of Medical Biophysics, University of Toronto, Toronto, ON Canada; 20 Hurvitz Brain Sciences Program, Sunnybrook Research Institute, Toronto, ON Canada; 21 Life and Health Sciences Research Institute (ICVS), School of Medicine, University of Minho, Braga, Portugal; 22 ICVS/3B’s, PT Government Associate Laboratory, Braga/Guimarães, Portugal; 23 2CA-Braga Cinical Academic Center, Hospital de Braga, 4710-243 Braga, Portugal; 24 Department of Psychiatric Research, Diakonhjemmet Hospital, Oslo, Norway; 25 Norwegian Centre for Mental Disorders Research (NORMENT), Institute of Clinical Medicine, University of Oslo, Oslo, Norway; 26 Laboratory of Surgical Neuroanatomy, Universitat de Barcelona, Barcelona, Spain; 27 Departamento de Psicología Básica, Clínica y Psicobiología, Universitat Jaume I, Castelló de la Plana, Spain; 28 Research Department, Vestre Viken Hospital Trust, 3004 Drammen, Norway; 29 Department of Psychology, University of Oslo, P. O. box 1094, Blindern 0317 Oslo, Norway; 30 Menninger Department of Psychiatry and Behavioral Sciences, Baylor College of Medicine, Houston, TX USA; 31 Turner Institute for Brain and Mental Health and School of Psychological Sciences, Monash University, Melbourne, VIC Australia; 32 Department of Radiology, Marqués de Valdecilla University Hospital, Valdecilla Biomedical Research Institute IDIVAL, Santander (Cantabria), Spain; 33 Advanced Computing and e-Science, Instituto de Física de Cantabria (UC-CSIC), Santander (Cantabria), Spain; 34 Institut d’Investigacions Biomèdiques August Pi i Sunyer (IDIBAPS), CIBERSAM, University of Barcelona, Barcelona, Spain; 35 Department of Psychiatry, University of Oxford, Oxford, UK; 36 Imaging Genetics Center, Mark & Mary Stevens Neuroimaging & Informatics Institute, Keck School of Medicine, University of Southern California, Marina del Rey, CA USA; 37 Centre for Psychiatry Research, Department of Clinical Neuroscience, Karolinska Institute & Stockholm Health Care Services, Stockholm Region, Stockholm, Sweden; 38 Highfield Unit, Warneford Hospital, Oxford, UK

**Keywords:** Neuroscience, Psychiatric disorders

## Abstract

**Introduction:**

Regional gray matter (GM) alterations have been reported in early-onset psychosis (EOP, onset before age 18), but previous studies have yielded conflicting results, likely due to small sample sizes and the different brain regions examined. In this study, we conducted a whole brain voxel-based morphometry (VBM) analysis in a large sample of individuals with EOP, using the newly developed ENIGMA-VBM tool.

**Methods:**

15 independent cohorts from the ENIGMA-EOP working group participated in the study. The overall sample comprised T1-weighted MRI data from 482 individuals with EOP and 469 healthy controls. Each site performed the VBM analysis locally using the standardized ENIGMA-VBM tool. Statistical parametric T-maps were generated from each cohort and meta-analyzed to reveal voxel-wise differences between EOP and healthy controls as well as the individual-based association between GM volume and age of onset, chlorpromazine (CPZ) equivalent dose, and other clinical variables.

**Results:**

Compared with healthy controls, individuals with EOP showed widespread lower GM volume encompassing most of the cortex, with the most marked effect in the left median cingulate (Hedges’ *g* = 0.55, *p* = 0.001 corrected), as well as small clusters of lower white matter (WM), whereas no regional GM or WM volumes were higher in EOP. Lower GM volume in the cerebellum, thalamus and left inferior parietal gyrus was associated with older age of onset. Deficits in GM in the left inferior frontal gyrus, right insula, right precentral gyrus and right superior frontal gyrus were also associated with higher CPZ equivalent doses.

**Conclusion:**

EOP is associated with widespread reductions in cortical GM volume, while WM is affected to a smaller extent. GM volume alterations are associated with age of onset and CPZ equivalent dose but these effects are small compared to case-control differences. Mapping anatomical abnormalities in EOP may lead to a better understanding of the role of psychosis in brain development during childhood and adolescence.

## Introduction

Early-onset psychosis (EOP) is defined as psychosis with onset before the age of 18; this includes early-onset schizophrenia (EOS), affective and other non-affective psychotic disorders. EOP occurs during a critical period of neurodevelopment and neuromaturation and is associated with adverse long-term outcomes, including more severe and long-lasting symptoms and less response to treatment. A long-term study reported that 60% of individuals with EOP had poor outcomes at their 40-year follow-up, measured with the Global Assessment Scale [1]. Furthermore, compared to other psychoses, EOP has a generally poorer outcome [2], indicating a more severe illness course. The increased level of psychopathology in EOP suggests that the brain could be affected to a greater degree than in adult-onset psychosis [3, 4].

Due to the low prevalence of EOP, many imaging studies in this patient group are limited in sample size and statistical power. The Enhancing Neuro Imaging Genetics through Meta-Analysis initiative (ENIGMA; http://enigma.ini.usc.edu) addresses this issue by pooling samples from research groups around the world. The ENIGMA EOP Working Group has previously analyzed subcortical regions using FreeSurfer (https://surfer.nmr.mgh.harvard.edu) from 263 EOP and 359 healthy controls (HC) and found that EOP was associated with significantly lower intracranial volume and hippocampal volume. The study also reported that individuals with EOP had higher caudate, and pallidum volumes [5].

FreeSurfer analyses are typically based on anatomically defined regions of interest (ROIs); in contrast, a different analysis technique known as Voxel-Based Morphometry (VBM) examines volumes in hundreds of thousands of voxels throughout the entire brain. This may provide complementary information to FreeSurfer by localizing focal abnormalities within an anatomically defined region [6]. Previous meta-analyses based on VBM data have found gray matter (GM) abnormalities in schizophrenia as well as in EOS [7–10]. However, these meta-analyses all used published coordinates from studies -these publications are limited as they only take into account significant peak findings and ignore sub-threshold results. In addition, the studies included in these meta-analyses varied in their methodology, including different registration and segmentation methods, amounts of smoothing and selection of covariates in the statistical analysis. To address these issues, we designed a new VBM pipeline for the ENIGMA consortium (hereafter referred to as the ENIGMA-VBM tool) that processes images in a standardized way and conducts automatic quality control. The tool is designed so that each cohort in the ENIGMA consortium processes their own sample using the ENIGMA VBM tool and sends their result to the coordinating center for final analysis. This has the added advantage that sites have the option of only sharing group data if required for data privacy and ethical permission. In this study, we introduce the ENIGMA VBM Tool, and present the largest VBM analysis to date of individuals with EOP. Our main aim was to map gray and white matter (WM) volume difference across the whole brain in EOP and examine the role of clinical variables such as age of onset, duration of illness, and chlorpromazine (CPZ) equivalent doses. In sensitivity analyses we also planned to test how robust the results were to changes in VBM processing parameters. In this study, we expected lower cortical GM volume in EOP in frontal and temporal regions, and based on the previous FreeSurfer ENIGMA EOP study [5] we also expected to find smaller volumes of the hippocampus and increased volume in the striatum.

## Methods

### Cohorts and Participants

The sample comprised 482 individuals with EOP and 469 HC from 15 independent cohorts from the ENIGMA-EOP working group. Diagnoses were established according to either the Diagnostic and Statistical Manual of Mental Disorders (DSM-IV or DSM-V) or the International Classification of Diseases (ICD-10). Details of each cohort are shown in supplementary Table S1; cohort-wise inclusion and exclusion criteria are shown in supplementary Table S2. In addition to diagnosis, sex and age, cohorts collected clinical data from each patient. The following clinical data was recorded by at least 5 cohorts: age of onset of psychosis, positive and negative syndrome scale [PANSS [11]] and current chlorpromazine (CPZ) equivalent dose, calculated according to Woods et al. [12]; full scale IQ was also measured. All study participants or their legal guardians provided written informed consent and each cohort received approval from their local ethics committee. The study was conducted in accordance with the Declaration of Helsinki.

### ENIGMA-VBM tool

The ENIGMA-VBM tool (https://sites.google.com/view/enigmavbm) was specifically developed for the ENIGMA consortium by the authors. The tool processes case and control 3D T1-weighted brain images from each cohort using a DARTEL (Diffeomorphic Anatomical Registration Through Exponentiated Lie Algebra) VBM processing pipeline in SPM12 (Statistical Parametric Mapping; https://www.fil.ion.ucl.ac.uk/spm/) [13]. The software requests clinical and demographic data in a standardized text file and then conducts voxel-wise statistical analysis for gray and white matter and outputs three-dimensional statistical T-maps. The T-maps are then meta-analyzed to determine case-control differences for all cohorts combined using the ‘Permutation of Subject Images’ version of Seed-based d-Mapping (SDM-PSI, v6.21, https://www.sdmproject.com) [14]. SDM is a voxel-based meta-analytic software, developed to pool data from published coordinates or T-maps. The VBM tool also conducts regression analyses of clinical variables in the patient group (e.g., examining where in the brain changes in GM are associated with symptom severity) within each cohort. This approach has greater statistical power than a meta-regression, as the analysis is completed within the cohort and then pooled, rather than computing a mean value of a clinical variable for each cohort. In the previous literature, VBM analyses have used various smoothing kernel sizes and different combinations of covariates. To examine how robust the results are to changes in these parameters, the ENIGMA-VBM tool conducts one main standard analysis for gray and white matter (smoothing=8 mm, covariates=Intracranial volume [ICV] and age) which was fixed a priori and also conducts sensitivity analyses by varying the smoothing kernel from 2 mm to 12 mm and applying specific combinations of covariates. The tool also tests the differences between modulated and non-modulated process. Modulation adjusts the volume change caused by the normalization step in VBM, ensuring that volume is preserved when areas of the brain are compressed or dilated to match the MNI template image and has been shown to reveal changes that unmodulated processing fails to show [15]. The sensitivity analyses are to test whether our results are robust to changes in VBM processing parameters, and in addition they may provide additional information on the optimal parameters for future VBM studies. Further information is available in the supplementary materials.

### Image processing and analysis

T1-weighted brain imaging data from individuals with EOP and HC was acquired at each participating cohort. Cohort-wise scanner and acquisition information is shown in Supplementary Table S3. Each cohort used the ENIGMA-VBM tool v1.013 to examine gray and white matter volume in cases compared to controls and examined the effect of clinical variables in the patient group.

The ENIGMA-VBM tool generated T-maps for each cohort for each analysis. These maps include the result of statistical tests at each cohort for approximately 200,000 voxels across the brain. The covariates of no interest for each of the T-maps were a) ICV and age for the case-control comparison and b) ICV, age and sex for the clinical variables, in the EOP group. We chose ICV and age as covariates for the main analysis as these variables account for the most variance in segmented GM and WM data, we added sex for clinical variables because of concerns that sex may associate with particular clinical variables (e.g., age of onset). For each analysis, T-maps from each cohort were pooled using SDM. In this project we used T-maps as inputs instead of collecting coordinates from sites. Briefly, the SDM software pipeline performs each of the following steps: 1) converts cohort T-maps into effect size maps using standard formulas, 2) meta-analyzes effect size maps with a random-effects model, 3) applies family-wise error (FWE) correction for multiple comparisons using threshold-free cluster enhancement (TFCE) with statistical thresholding (p < 0.025, voxel extent ≥10) [16].

There were 2 cohorts where EOP individuals were scanned after 18 years old which could potentially cause difference in results. We therefore performed an additional analysis including only 13 cohorts that included individuals who were scanned before 18. We were interested in clinical subtypes - EOS, affective psychosis (AFP) and other psychosis (OTP), however as individual data was not transferred by the tool, we could not make direct group comparisons. To examine cohorts with a majority of EOS individuals, we excluded 9 cohorts that included AFP and repeated the case-control comparison. To further explore clinical subtypes, two meta-regressions were performed using the proportion of EOS and AFP in each cohort, respectively, as a regressor.

Mean and standard deviations (SD) for global GM and WM in litres were generated by the ENIGMA-VBM tool run at each site, for the EOP and HC groups. These unadjusted values were pooled using a random-effects model in STATA (StataCorp. 2021. *Stata Statistical Software: Release 17*. College Station, TX: StataCorp LLC).

The study included several analyses. To deal with multiple comparisons with each analysis, we provided FWE results. The primary aim of the study was the case-control analysis of gray and white matter which was chosen a priori. The analysis of clinical variable should be considered as exploratory, while the sensitivity analyses verify that the main analysis survives variations in the methodology.

## Results

All voxel-wise data from the analyses below are available to download from Neurovault (https://neurovault.org/collections/KUZFROFC/). Reported *p*-values quoted for voxel-wise neuroimaging data are FWE-corrected for multiple comparisons using TFCE.

### Demographics and clinical information

Demographics and clinical characteristics from each cohort are shown in Table 1. Individuals with EOP were diagnosed with psychosis before age 18 and were aged 14 to 26 when scanned, combined mean age at scan was 17.1 and age of onset was 15.3. All cohorts included data on age, sex, and diagnosis. In terms of additional clinical data, 14 cohorts included age of onset data, 13 cohorts included duration of illness data (mean=1.9 years), 10 cohorts included data for CPZ equivalent dose (mean=234.2), 9 cohorts included the PANSS score (mean PANSS pos=17.5, neg=15.5), and 6 cohorts included full scale IQ (mean=96).

**Table 1:** Cohort-wise demographic and clinical information.

Cohort	Number of participants		Diagnosis (EOS/AFP/OTP)	Female N (%)		Mean Age ± SD		EOP specific measures Mean ± SD						Scanner field strength (Tesla)
	EOP	HC		EOP	HC	EOP	HC	Age of onset^a^	Duration of illness^b^	IQ^c^	PANSS Neg.^d^	PANSS Pos.^d^	CPZ^e^	3.0
**ROME**	29	14	23/1/5	8 (27.6)	8 (57.1)	16.8 ± 2.0	13.6 ± 1.7	15.7 ± 2.3	1.1 ± 0.9	NA	15.9 ± 5.7	20.2 ± 7.9	375.8 ± 735.7	3.0
**SCAPS**	34	23	2/20/12	18 (52.9)	18 (78.3)	16.4 ± 1.2	16.7 ± 1.3	15.0 ± 2.0	1.5 ± 1.6	NA	NA	NA	166.7 ± 146.2	3.0
**RUND**	22	31	15/0/7	11 (50.0)	15 (48.4)	15.9 ± 1.7	15.5 ± 1.7	14.4 ± 2.0	1.9 ± 1.4	96.8 ± 14.0	11.5 ± 4.8	14.3 ± 4.2	174.5 ± 226.5	1.5
**YTOP-1**	22	30	15/0/7	15 (68.2)	18 (60.0)	16.7 ± 1.1	16.3 ± 1.3	14.6 ± 2.0	2.1 ± 1.9	102.1 ± 11.8	20.5 ± 7.2	18.2 ± 3.9	136.3 ± 232.9	3.0
**YTOP-2**	18	40	8/2/8	15 (83.3)	21 (52.5)	16.2 ± 1.2	16.1 ± 1.6	14.8 ± 1.6	1.3 ± 0.8	97.4 ± 13.7	17.2 ± 6.4	15.1 ± 4.8	159.7 ± 250.8	3.0
**KCL-1**	14	15	14/0/0	4 (28.6)	6 (40.0)	16.8 ± 1.4	16.2 ± 1.7	14.5 ± 2.4	2.2 ± 1.6	97.4 ± 11.3	13.6 ± 3.0	10.9 ± 3.8	NA	1.5
**KCL-2**	20	25	6/2/12	0 (0)	0 (0)	17.0 ± 1.2	16.4 ± 1.6	16.3 ± 1.4	1.1 ± 0.8	NA	13.5 ± 6.7	15.4 ± 7.1	NA	3.0
**OXFORD**	44	24	44/0/0	17 (38.6)	10 (41.7)	16.3 ± 1.3	16.0 ± 1.5	14.6 ± 1.6	1.8 ± 1.3	89.2 ± 15.9	16.0 ± 3.2	22.4 ± 2.8	345.6 ± 236.1	3.0
**BARCELONA-1**	48	23	33/11/4	22 (45.8)	12 (52.2)	16.3 ± 1.6	16.6 ± 1.2	15.9 ± 1.6	0.5 ± 0.7	NA	13.9 ± 6.5	21.0 ± 7.0	264.1 ± 228.7	1.5
**BARCELONA-2**	120	78	42/64/14	60 (50.0)	48 (61.5)	16.0 ± 1.5	15.7 ± 2.1	15.6 ± 1.6	0.4 ± 0.5	NA	17.1 ± 7.1	19.9 ± 6.6	257.5 ± 178.2	3.0
**PAFIP**	17	12	17/0/0	7 (41.2)	4 (33.3)	18.0 ± 0.6	17.5 ± 1.3	17.6 ± 0.6	0.4 ± 0.3	93.2 ± 14.1	NA	NA	185.0 ± 83.0	3.0
**SRI**	28	75	0/28/0	16 (57.1)	38 (50.7)	17.5 ± 1.3	16.9 ± 1.8	15.4 ± 2.2	2.2 ± 2.0	NA	NA	NA	NA	3.0
**MADRID**	28	57	26/0/2	6 (21.4)	27 (47.4)	15.9 ± 1.7	12.0 ± 3.2	NA	NA	NA	NA	NA	NA	1.5
**SYDNEY**	26	10	11/3/12	8 (30.8)	8 (80.0)	22.0 ± 4.4	18.4 ± 3.2	14.7 ± 2.3	7.9 ± 4.8	NA	NA	NA	276.7 ± 237.6	3.0
**FEMS**	12	12	0/12/0	2 (16.7)	3 (25.0)	19.4 ± 1.2	19.8 ± 2.1	16.8 ± 1.1	NA	NA	NA	NA	NA	3.0
**Combined total or mean**	482	469	256/143/83	209 (43.4)	236 (50.3)	17.1	16.2	15.3	1.9	96.0	15.5	17.5	234.2	

*EOP* early-onset psychosis, *EOS* early-onset schizophrenia, *AFP* affective psychosis, *OTP* other psychosis, *HC* healthy controls, *PANSS* Positive and Negative Syndrome Scale, *IQ* intelligence quotient, *CPZ* chlorpromazine equivalent medication dose (mg), NA indicates the data was not available.

^a^Age of onset data is available from 439 patients from 14 cohorts.

^b^Duration of illness data is available from 427 patients from 13 cohorts.

^c^IQ data is available from 141 patients from 6 cohorts.

^d^PANSS data is available from 311 patients from 9 cohorts.

^e^CPZ data is available from 342 patients from 10 cohorts.

### Case-control differences in regional and global gray and white matter volume

**Fig. 1:**
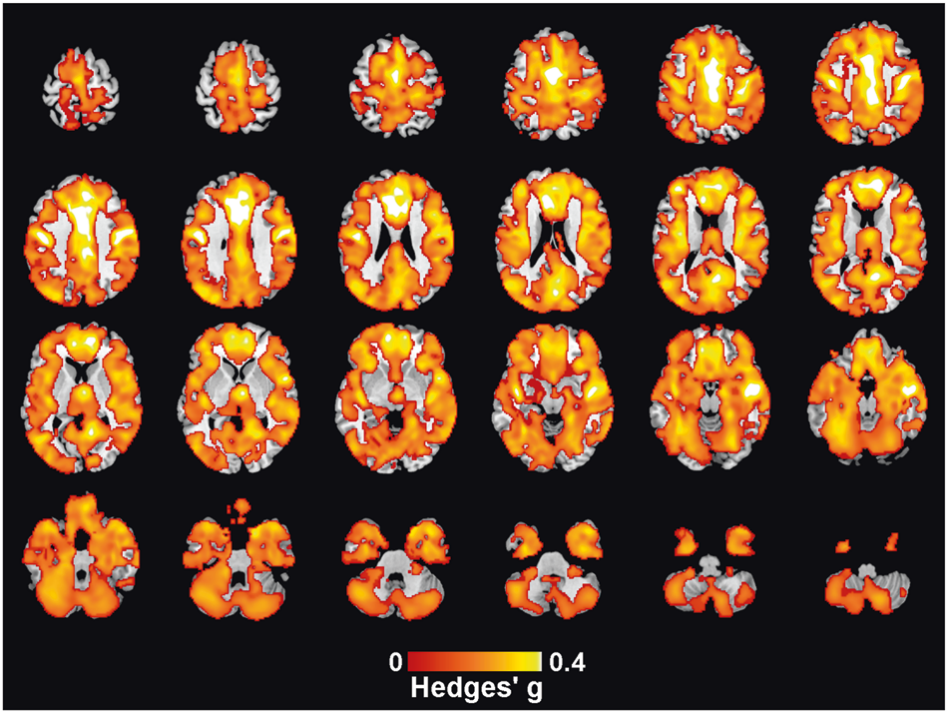
Regions showing lower GM volume in individuals with EOP compared to HC. This analysis controls for age and ICV. Peak MNI coordinate [-4,-4,48], Hedges’ *g* = 0.55. Bilateral median cingulate, bilateral anterior cingulate, right middle temporal gyrus and right postcentral gyrus all have Hedges’ *g* > 0.4, see Table S4.

Regional and global case-control analyses included data from all 15 cohorts including 482 individuals with EOP and 469 HC. Compared with HC, widespread lower GM volume was observed in individuals with EOP in all lobes of the brain with a peak effect in the median cingulate (Hedges’ *g* = 0.55, *p* = 0.001). The large cluster also comprised temporal, frontal and postcentral gyri (Fig. 1 and Table S4). No regional increase in GM volume was identified. The analysis including 13 cohorts where individuals were scanned before age 18 showed similar result (Fig. S1 and Table S5), with a peak effect size of Hedges’ *g* = 0.53 (*p* = 0.001).

Individuals with EOP had small clusters of lower WM volume in the bilateral inferior longitudinal fasciculus (Fig. S2 and Table S6). There were no detectable WM increases in the EOP group compared to the HC.

**Fig. 2:**
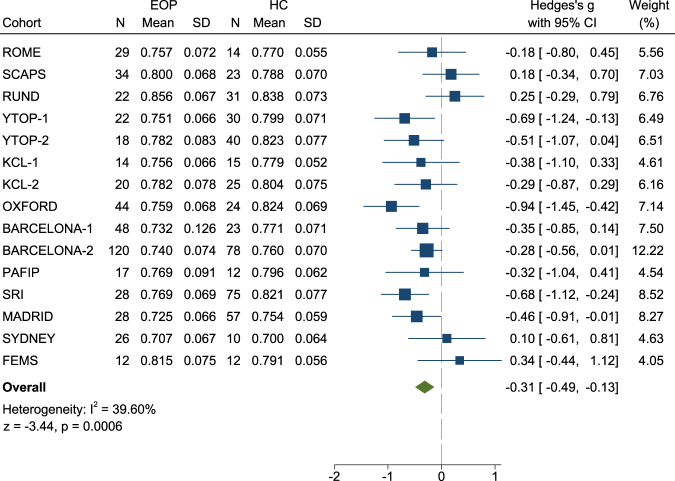
Forest plot of studies included in the meta-analysis of global gray matter differences between EOP and HC.

Individuals with EOP had significantly lower *global* GM volume (Fig. 2), Hedges’ *g* = -0.31 (95% CI -0.49 to -0.13, *p* = 0.0006). There was no significant difference between individuals with EOP and controls in global WM volume (Fig. S3).

### Regional volume changes associated with clinical variables

**Fig. 3:**
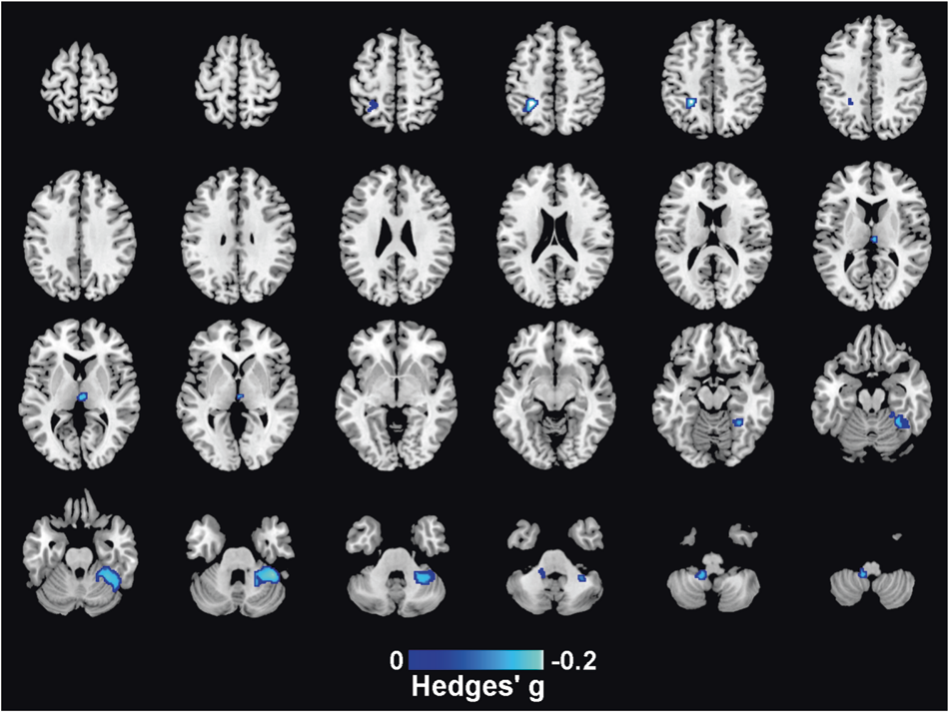
Regions showing lower GM volume associated with an older age of onset among EOP individuals. This analysis controls for age, ICV and sex. Peak MNI coordinate [40,-42,-30], Hedges’ *g* = -0.20.

Age of onset was available from 14 cohorts including 439 individuals with EOP. An older age of onset in EOP was associated with lower GM volume in the right cerebellum (Hedges’ *g* = -0.20, *p* = 0.002), left cerebellum (Hedges’ *g* = -0.17, *p* = 0.015), left inferior parietal gyri (Hedges’ *g* = -0.24, *p* = 0.002), and thalamus (Hedges’ *g* = -0.17, *p* = 0.022), shown in Fig. 3 and Table S7. An older age of onset in EOP was associated with lower WM volume in middle cerebellar peduncles (Hedges’ *g* = -0.18, *p* = 0.012), shown in Fig. S4 and Table S8. We did not find any regions where a younger age of onset was associated with lower GM or WM volume.

**Fig. 4:**
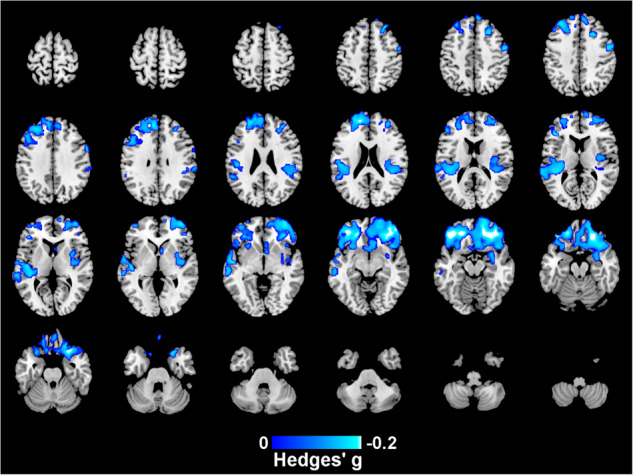
Regions showing lower GM volume associated with a higher chlorpromazine (CPZ) equivalent dose in EOP individuals. This analysis controls for age, ICV and sex, peak MNI coordinate [-40,36,-18], Hedges’ g = -0.24.

CPZ equivalent dose analyzed in SDM were obtained from 10 cohorts including 342 individuals with EOP. Widespread lower GM volume in multiple regions was associated with a higher CPZ equivalent dose (Hedges’ g = -0.24, *p* = 0.001), see Fig. 4 and Table S9. We did not identify any regions of increased GM volume associated with increased CPZ equivalent dose.

Longer duration of illness was associated with higher right precentral and fusiform gyrus volumes (Fig. S5 and Table S10). Higher IQ was associated with higher GM volume in biliteral temporal regions, left cuneus cortex, and other small clusters (Fig. S6 and Table S11).

The meta-analysis excluding individuals with AFP included 147 EOP and 169 HC (from 6 cohorts) and revealed similar results to the main meta-analysis highlighting a peak difference in left median cingulate, but with a larger effect size (Hedges’ g = 0.72, p < 0.001, Fig. S7 and Table S12). Both meta-regressions that used the proportion of EOS and AFP as a predictor did not show significant results.

### Sensitivity analysis

GM volume differences between EOP and HC, controlling for different combinations of covariates (age, sex, ICV and total GM volume), are shown in Figs. S8–S13 and Table S13-S18, while equivalent WM volume analyses are shown in Fig. S14–S18 and Table S19-S23. The GM results were relatively insensitive to different covariates with the exception of adjusting for total GM (rather than ICV) which revealed larger volumes in the striatum bilaterally in EOP individuals (Fig. S9 and Table S14). The WM results were also insensitive to different covariates, however including no covariates or adjusting for total WM resulted in less volumetric group differences (Figs. S15, S18 and Table S20, S23). In Figs. S19–S20 and Table S24-S25, we provide analysis using non-modulated data for GM and WM. Non-modulated data showed greater volume differences for GM corresponding with stronger effect sizes, and a different pattern of changes for WM. GM volume analyses using 2 mm, 4 mm, 8 mm, 12 mm smoothing kernels are shown in Fig. S21 and Table S26; the results show more spatially extensive GM changes as the smoothing kernel increases in size. WM analyses using different smoothing kernels are shown in Fig. S22 and Table S27. The heterogeneity for all analyses quantified by I^2^ was generally low, with mean I^2^ of 14.52% for regional GM volume differences between EOP and HC (Fig. S23).

## Discussion

In this study, we found significantly lower regional GM volumes in individuals with EOP compared to HC across most of the cerebral cortex and cerebellum. The peak reduction was in the left median cingulate with a robust effect size of Hedges’ g = 0.55 with subpeaks in other areas of the cingulate, right middle temporal gyrus and postcentral gyrus. Additionally, in the primary case-control analysis we found no regions where GM volume was higher in individuals with EOP compared to HC. These findings were consistent with the global analysis with lower total GM in EOP. In contrast, effects on WM were minimal with only small clusters of lower volume identified, the largest of which was in the right longitudinal fasciculus. The association with clinical variables consistently had lower effect sizes than the case-control comparison. Those who developed EOP at a later age had lower GM volume in a number of small regions compared to those with an earlier age of onset. In addition, those who received higher doses of antipsychotic medication had lower GM volumes in the frontal and temporal cortex.

### Regional and total brain volume

In EOP, the most pronounced region with lower GM volume was found in the cingulate cortex, followed by regions in the frontal and temporal cerebral cortex. We also found lower volumes in the bilateral insula, thalamus, fusiform gyrus, hippocampus, and parahippocampal regions, however these regions have smaller effect sizes. Interestingly, the GM findings were widespread even though ICV was controlled for. The finding of lower GM volume in frontal, temporal and parietal cortices in individuals with EOP is supported by previous studies [17], and also wider schizophrenia studies [8]. Gurholt et al. [5] also used data from the ENIGMA-EOP working group but used ROI-based subcortical volumes from FreeSurfer rather than VBM; they also reported lower hippocampal volume but also found higher caudate and pallidum volume while our result showed no higher GM volume in any region. The differences are likely to be due to the methodology used, as most FreeSurfer analyses examine pre-defined anatomical regions, whereas VBM examines changes in thousands of voxels across the brain. As there are also several differences in the pre-processing steps, the methods are likely to have different sensitivity to brain abnormalities and some researchers have suggested both methods should be used [18]. A previous VBM meta-analysis of 7 studies in early-onset schizophrenia [7] used a technique known as activation likelihood estimation (ALE) but found no significant volume reduction at *p* < 0.05 after FWE correction. This may have been because the ALE technique uses peak coordinates of clusters and does not consider sub-threshold data. Another recent coordinate SDM meta-analysis of 114 VBM studies in schizophrenia by Picó-Pérez et al. [19] also reported widespread lower GM volume throughout the brain. The peak coordinate reported by Picó-Pérez et al. was the left rolandic operculum followed by other regions in the temporal and frontal GM. While the meta-analysis by Pico-Perez et al. could possibly include individuals with EOP, demographic data from the individual studies suggest these were nearly all adult onset. The authors of Pico-Perez et al. shared with us their Hedges’ g map showing the effect size of GM reductions across the brain which we were able to compare to our findings (Fig. S24). Qualitatively comparing the results, the adult individuals with schizophrenia had greater GM decrease in both temporal lobes, while EOP had greater volume reductions around the cingulate region. However, as Pico-Perez et al. used published peak coordinates and we used T-maps, some caution should be taken when comparing these findings. These differences in brain volume tentatively suggest that the cingulate may be more extensively affected in EOP. Longitudinal studies of individuals with EOP and individuals with schizophrenia would help to clarify if these abnormalities are fixed at illness onset or evolve over time.

Considering WM, our results showed bilateral small clusters with lower WM volume in the inferior longitudinal fasciculus, cingulum and corpus callosum. We are not aware of any previous VBM meta-analysis that examined WM volume changes in EOP. Haijma et al. [20] found a significant reduction in total WM volume in a large region-of-interest meta-analysis and the reported change was much smaller than they observed in GM, which is consistent with our results. Smaller postmortem studies in schizophrenia have not found significant differences in WM volume between schizophrenia subjects and healthy controls [21, 22]. A key question for the pathophysiology of psychosis is why GM is affected more than WM. GM primarily includes neuronal cell bodies while WM includes myelinated axons and volume reductions in the different tissues may reflect a pathophysiology that affects different parts of the neuron [23]. It has been hypothesized that WM myelin dysfunction in schizophrenia is a secondary effect to synaptic dysfunction, so the WM loss occurs after GM loss [24]. In a neurodevelopmental aspect, WM maturation does not stop until after 30 [25, 26], so it is conceivable that WM could adapt or ‘normalize’ after the onset of psychosis.

### Association with clinical variables

An older age of onset in individuals with EOP was associated with lower GM volume in the inferior parietal gyrus, thalamus and cerebellum. In addition, a longer duration of illness was associated with greater volumes of the right precentral and fusiform gyrus. Given that individuals with an earlier age of onset have a generally poorer outcome [1], we expected that a younger age of onset [27] and longer duration of illness [20, 28] would be associated with lower GM. However, the findings were in the opposite direction. More recent studies have not found an association between GM volume and age of onset [5, 29, 30]. Studies in healthy volunteers have shown that GM volume in the frontal, temporal and parietal lobes peaks during early adolescence, and then decreases [31]. For individuals with an early age of onset, emergence of psychosis may coincide with the GM peak, and interfere with the normal subsequent reduction in GM volume. Another hypothesis is that the developmental pruning in the brain, which is associated with the development of new circuits of cognition and function, can be disrupted by psychosis [24, 32]. Thus, leading to findings of relatively increased regional GM volume in those with an earlier age of onset. Longer duration of illness may be confounded by the usage of antipsychotics, so the result should be interpreted with caution. Future longitudinal studies will help clarify how psychosis onset affects brain trajectories in adolescence.

In the present study, EOP individuals who received higher CPZ equivalent doses had lower GM volumes of the frontal, insula and precentral gyrus. A limitation of this analysis is that the CPZ data reflects current use as we did not have access to data on past use or cumulative exposure to antipsychotic medication. In first episode schizophrenia, typical antipsychotics in comparison with drug-free individuals have shown lower GM in similar regions but higher GM in right lenticular nucleus [33]. In the previously published ENIGMA schizophrenia study, CPZ equivalent dose correlated with widespread cortical thinning with the strongest effect in frontal and temporal regions [28, 34], and this finding is supported by longitudinal MRI studies [35, 36]. The mechanism of changes in brain structure from antipsychotic use is not known but this could be possibly linked to the association of reduced cerebral blood flow and antipsychotics [37]. In our study, we did not find an increase in basal ganglia volume associated with neuroleptics, as in other studies in schizophrenia [33, 35, 37, 38], which could possibly be a unique feature of EOP. A challenge with examining CPZ equivalent doses is that it is likely confounded by illness severity as individuals with more severe symptoms may be prescribed higher doses of antipsychotics.

### Sensitivity analysis

Voxel-Based Morphometry involves a number of processing steps including segmentation, normalization, modulation, smoothing and statistical analysis; variations in the parameters controlling these steps can all lead to different results [39]. An advantage of the ENIGMA-VBM tool is that all cohort data are processed using the same algorithm in order to achieve uniformity. However, in addition, the VBM tool also automatically conducts a series of sensitivity analyses to examine the effect of variations in the methodology, such as different covariates, and investigates the effects of different image preprocessing parameters. In VBM, smoothing is applied to reduce between-subject variations in brain anatomy, improve signal to noise, and increase the normality of the data. Different degrees of smoothing can be chosen, typically between 4 and 12 mm [40]. We found that with larger smoothing kernels, the results show more spatially extensive GM changes and the effect size also generally increased (Fig. S21). This is consistent with a previous study that reported larger smoothing kernels increased the spatial extent of significant clusters [39]. However, the disadvantage of large smoothing kernels is that the small abnormalities may be less accurately spatially determined due to the blurring effect of the kernel. A smaller smoothing kernel is more sensitive to small structural changes but may also increase false positive results [39, 41]. In our primary case and control comparison, we chose 8 mm as the default smoothing kernel to balance the specificity and sensitivity of results. Compared with modulated results, non-modulated results showed marginally greater effect sizes and an expanded region of GM (Fig. S19). Radua et al. [42] also have shown that VBM unmodulated analyses are more sensitive to cortical thinning than modulated analyses.

It is common to control for head size in VBM analyses by adjusting for total intracranial volume (ICV) or total GM volume. In the main analysis we controlled for ICV as this is the most common approach in VBM [3, 43, 44]. In contrast, controlling for total GM volume, one can examine which regional volumes are affected beyond group differences in total GM. When controlling for total GM volume (Fig. S9), we found smaller clusters of reduced volume and uniquely some regions with increased volume such as the striatum, which mirrored the previous FreeSurfer analysis of the ENIGMA data [5] showing increased volume of the caudate and pallidum. Of note, the striatum volume was higher in EOP compared to HC in the main ICV analysis but the finding was not significant. Adjusting for different covariates provides complementary information in the analyses and may also assist in selecting the optimal parameters to use in VBM for future studies.

The current study has some limitations. Although all EOP individuals had their first onset before 18, we were not aware whether all EOP individuals were first episode psychosis when they are scanned and how their condition fluctuated during their course, so the association between age of onset and GM needs to be interpreted with caution. We did not have data on how long patients had used antipsychotic medication, so the CPZ analysis indicates the association with current medication rather than with cumulative antipsychotic medication exposure. There were a small number of sites with significant difference in age between the patients and controls, however age was controlled for in the main analyses with an additional age squared term in the sensitivity analysis. For the analysis in SDM we used the whole brain mask rather than the GM or WM mask because a small number of voxels in globus pallidus were left out in the GM/WM mask; as striatal regions were of interest in psychosis we wanted to ensure all voxels were included. This may have influenced the results by having a more conservative multiple comparisons correction compared to studies using smaller GM or WM masks. All the studies included in the meta-analysis are case-control studies and so suffer from selection bias to a degree. However, including a large number of sites will likely reduce particular types of selection bias as each site will have their own method of recruitment. A previous review has suggested that while neuroimaging studies have potentially high levels of bias they were superior to case-control designs in other areas of psychiatry [45]. We found distinct structural alteration in EOP, but it is still unclear whether the brain structural changes arise from abnormal brain development before psychosis onset or as a consequence of illness, or both. Thus, longitudinal studies focusing on the prodromal phase or earlier during development may be able to better explain the development of psychosis.

In conclusion, we used the ENIGMA-VBM tool, a standardized VBM analysis framework to analyze GM and WM volume abnormalities in EOP in a large sample and provided robust evidence for distinct and widespread lower GM in EOP. The GM volume was also found to be influenced by age of onset, and medication but the effect sizes for these changes were smaller than the case-control comparison. Finally, we provide online the results of our analyses as 3D effect size maps for comparison to future meta-analyses.

### Supplementary information

Supplementary material A

Supplementary material B

## Data Availability

The datasets (statistical maps) generated during the current study are available from the NEUROVAULT repository, https://neurovault.org/collections/KUZFROFC/.
