# Supplementary material for: Mapping gray and white matter volume abnormalities in early-onset psychosis: an ENIGMA multicenter voxel-based morphometry study
Source: Mol Psychiatry. 2024 Jan 10;29(2):496–504. doi: 10.1038/s41380-023-02343-1 (PMC11116097; doi:10.1038/s41380-023-02343-1)

Table of Contents

[Table S1 Cohort overview 3](#_Toc145408370)

[Table S2 Cohort-wise inclusion and exclusion criteria 3](#_Toc145408371)

[Table S3 Scanner-specific image acquisition and processing details 13](#_Toc145408372)

[Table S4 Regional GM volume 15](#_Toc145408373)

[Figure S1 Regional GM differences from 13 cohorts scanned before age 18 16](#_Toc145408374)

[Table S5 Regional GM differences from 13 cohorts scanned before age 18 16](#_Toc145408375)

[Figure S2 Regional WM volume 18](#_Toc145408376)

[Table S6 Regional WM volume 18](#_Toc145408377)

[Figure S3 Global WM 19](#_Toc145408378)

[Table S7 Regional GM volume differences associated with age of onset 20](#_Toc145408379)

[Figure S4 Regional WM volume differences associated with age of onset 21](#_Toc145408380)

[Table S8 Regional WM volume differences associated with age of onset 21](#_Toc145408381)

[Table S9 Regional GM differences associated with CPZ equivalent dose 22](#_Toc145408382)

[Figure S5 Regional GM differences associated with duration of illness 22](#_Toc145408383)

[Table S10 Regional GM differences associated with duration of illness 23](#_Toc145408384)

[Figure S6 Regional GM differences associated with IQ 23](#_Toc145408385)

[Table S11 Regional GM differences associated with IQ 24](#_Toc145408386)

[Figure S7 Regional GM differences from 6 cohorts without AFP 25](#_Toc145408387)

[Table S12 Regional GM differences from 6 cohorts without AFP 25](#_Toc145408388)

[Regional GM differences analysis between EOP and HC controlling for different covariates 27](#_Toc145408389)

[Figure S8 GM controlling for Age, ICV and sex 27](#_Toc145408390)

[Table S13 GM controlling for Age, ICV and sex 27](#_Toc145408391)

[Figure S9 GM controlling for Age and total GM volume 29](#_Toc145408392)

[Table S14 GM controlling for Age and total GM volume 29](#_Toc145408393)

[Figure S10 GM controlling for ICV only 30](#_Toc145408394)

[Table S15 GM controlling for ICV only 30](#_Toc145408395)

[Figure S11 GM using Proportional scaling, controlling for age and ICV 31](#_Toc145408396)

[Table S16 GM using Proportional scaling, controlling for age and ICV 31](#_Toc145408397)

[Figure S12 GM controlling for age, age squared, ICV, sex 32](#_Toc145408398)

[Table S17 GM controlling for age, age squared, ICV, sex 32](#_Toc145408399)

[Figure S13 GM not controlling for any covariate 33](#_Toc145408400)

[Table S18 GM not controlling for any covariate 33](#_Toc145408401)

[Regional WM differences analysis between EOP and HC controlling for different covariates 34](#_Toc145408402)

[Figure S14 WM controlling for Age, ICV, sex 34](#_Toc145408403)

[Table S19 WM controlling for Age, ICV, sex 34](#_Toc145408404)

[Figure S15 WM controlling for Age and total WM volume 35](#_Toc145408405)

[Table S20 WM controlling for Age and total WM volume 35](#_Toc145408406)

[Figure S16 WM controlling for ICV 36](#_Toc145408407)

[Table S21 WM controlling for ICV 36](#_Toc145408408)

[Figure S17 WM using Proportional scaling, controlling for age and ICV 37](#_Toc145408409)

[Table S22 WM using Proportional scaling, controlling for age and ICV 37](#_Toc145408410)

[Figure S18 WM not controlling for any covariate 38](#_Toc145408411)

[Table S23 WM not controlling for any covariate 38](#_Toc145408412)

[Figure S19 GM volume analysis with no modulation in the VBM image processing and no covariates 39](#_Toc145408413)

[Table S24 GM volume analysis with no modulation in the VBM image processing and no covariates 39](#_Toc145408414)

[Figure S20 WM volume analysis with no modulation in VBM image processing and no covariates 40](#_Toc145408415)

[Table S25 WM volume analysis with no modulation in VBM image processing and no covariates 40](#_Toc145408416)

[Figure S21 Regional GM volume difference between EOP and HC using different smooth kernels (2mm, 4mm, 8mm, 12mm) 41](#_Toc145408417)

[Table S26 Regional GM volume difference between EOP and HC using different smooth kernels (2mm, 4mm, 8mm, 12mm) 42](#_Toc145408418)

[Figure S22 Regional WM volume difference between EOP and HC using different smooth kernels (2mm, 4mm, 8mm, 12mm) 43](#_Toc145408419)

[Table S27 Regional WM volume difference between EOP and HC using different smooth kernels (2mm, 4mm, 8mm, 12mm) 44](#_Toc145408420)

[Figure S23 Heterogeneity of effect size measured by I^2^ statistics (GM EOP vs. HC) 46](#_Toc145408421)

[Figure S24 Comparison of regional GM volume in EOP and Schizophrenia 47](#_Toc145408422)

## Table S1 Cohort overview

| **Cohort Name** | **PI** | **Institution (at time of recruitment)** | **City** | **Country** |
| --- | --- | --- | --- | --- |
| **ROME** | Gianfranco Spalletta | IRCCS Santa Lucia Foundation | Rome | Italy |
| **SCAPS** | Ingrid Agartz, Mathias Lundberg | Karolinska Institutet | Stockholm | Sweden |
| **RUND** | Bjørn Rishovd Rund | University of Oslo | Oslo | Norway |
| **YTOP-1** | Ingrid Agartz | University of Oslo | Oslo | Norway |
| **YTOP-2** | Ingrid Agartz | University of Oslo | Oslo | Norway |
| **KCL-2** | Anne-Kathrin J. Fett | University of London | London | UK |
| **OXFORD** | Anthony James | University of Oxford | Oxford | UK |
| **BARCELONA-1** | Josefina Castro-Fornieles, Gisela Sugranyes | Hospital Clínic i Provincial | Barcelona | Spain |
| **BARCELONA-2** | Josefina Castro-Fornieles, Gisela Sugranyes | Hospital Clínic i Provincial | Barcelona | Spain |
| **PAFIP** | Benedicto Crespo-Facorro | Hospital Universitario Marqués de Valdecilla | Santander | Spain |
| **SRI** | Benjamin I. Goldstein | Sunnybrook Health Science Center | Toronto | Canada |
| **MADRID** | Celso Arango | Hospital General Universitario Gregorio Marañón | Madrid | Spain |
| **SYDNEY** | Ian Hickie | University of Sydney | Sydney | Australia |
| **KCL-1** | Sophia Frangou | King’s College London | London | UK |
| **FEMS** | Michael Berk | Deakin University | Geelong | Australia |

## Table S2 Cohort-wise inclusion and exclusion criteria

| **Cohort** | **Diagnosis** | **Instrument for clinical assessment** | **Recruitment information** | **Inclusion criteria** | **Exclusion criteria** |
| --- | --- | --- | --- | --- | --- |
| **ROME** | EOP | DSM-V psychiatric and personality disorders using the SCID-5-RV and SCID- 5-PD | Local catchment area and referral from adolescent psychiatric units | Age between 10-18 years; onset of first psychotic positive symptom within a psychotic episode before age of 18; suitability for MRI scanning; written informed consent | History of alcohol or drug abuse in the two years before the assessment; lifetime drug dependence; traumatic head injury with loss of consciousness; past or present major medical illness or neurological disorders; intellectual disability; pervasive developmental disorder |
|  | HC | Comprehensive Assessment of Symptoms and History |  | Matched to patients (age, sex, laterality index, drug history, years of education); written informed consent | Current or past history of psychiatric, neurological or general medical illnesses, including substance dependence and significant loss of consciousness; presence of psychosis in first-degree relatives |
| **SCAPS** | EOP | DSM-IV | Specialist care unit of psychosis and bipolar disorder in the department of Child and Adolescent Psychiatry in Stockholm. Sweden | Psychotic disorders included: schizophrenia; schizoaffective disorder; psychotic depression; unspecified psychosis; bipolar I and II disorder; age between 12-18 years | Substance-induced psychotic disorder; IQ < 70; previous moderate to severe head injury; organic brain disease |
|  | HC | NA | Invitation by letter after random draw from the Swedish National Registry | Age between 12-18 years. good command of the Swedish language to complete interview and neurocognitive tests | History of mental health issues (contact with specialist services); previous or current use of psychotropic medication; first degree relatives with a history of psychotic disorders; IQ < 70; previous moderate to severe head injury; organic brain disease |
| **RUND** | EOP | SCID-I, mod A-D; PANSS; GAF Split version | Recruited from clinicians at in- and outpatient child- and adolescent mental health clinics in Southern Norway | Age 12-18. Broad schizophrenia spectrum disorder. | Psychosis NOS. History of head injury. IQ<70 |
|  | HC | Screened with MINI, screening mod | Recruited from schools in the patient catchment area, plus some from the database of the Norwegian Central Bureau of Statistics |  |  |
| **YTOP-1 & YTOP-2** | EOP | K-SADS-PL (2009)/ DSM-IV | In-and outpatient clinics for child and adolescent mental health in the greater Oslo area | Age between 12-18 years; diagnosis of psychotic disorder; good command of the Norwegian language to complete interview and neurocognitive tests | Substance-induced psychotic disorder; IQ < 70; previous moderate to severe head injury; organic brain disease |
|  | HC | K-SADS-PL (2009) | Invitation by letter after random draw from the Norwegian National Registry | Age between 12-18 years; good command of the Norwegian language to complete interview and neurocognitive tests | History of mental health issues (contact with specialist services); previous or current use of psychotropic medication; first degree relatives with a history of psychotic disorders; IQ < 70; previous moderate to severe head injury; organic brain disease |
| **KCL-2** | EOP | ICD-10 diagnosed by treating clinician | Patients were recruited via consultant psychiatrists and via the Mental Health Research Network (MHRN) in South London and Maudsley-, North East London- and South Essex Partnership University NHS Foundation Trusts. | Inclusion criteria for patients consisted of (1) age between 13-19 years, (2) experienced a psychotic episode according to ICD-10 criteria, as diagnosed by their clinician, (3) good command of the English language and (4) being able and willing to give written informed consent. |  |
|  | HC | No dx confirmed in telephone screening | Control participants were recruited from local schools, the Institute of Psychiatry volunteer database ‘Mindsearch’, via colleagues and previous participants. | Inclusion criteria for the control group were (1) age between 13-19 years, (2) good command of the English language and (3) able and willing to give written informed consent, and (4) no personal or family history of a psychotic illness. |  |
| **OXFORD** | EOP | KSADS-PL | Local adolescent psychiatric units | DSM IV Schizophrenia | Moderate mental impairment; a history of substance abuse or pervasive developmental disorder; significant head injury; neurological disorder or major medical disorder |
|  | HC | KSADS-PL | Local GP practices | Healthy adolescents | Moderate mental impairment; a history of substance abuse or pervasive developmental disorder; significant head injury; neurological disorder or major medical disorder |
| **BARCELONA-1 & BARCELONA 2** | EOP | K-SADS/ DSM-IV-TR | Referral from inpatient or outpatient units of the Department of Child and Adolescent Psychiatry and Psychology of the Hospital Clinic Barcelona | Age between 10-18 years; onset of first psychotic positive symptom within a psychotic episode before age of 18; diagnosis of a psychotic disorder per DSM-IVTR criteria; written informed consent | Intellectual disability per DSMIV-TR criteria (IQ < 70 & impaired functioning); pervasive developmental disorder; past history of head trauma with loss of consciousness; pregnancy |
|  | HC | K-SADS/ DSM-IV-TR | Local catchment area via advertisements | Age between 10-18 years; written informed consent | Past history of psychotic illness; current diagnosis of any Axis-I DSM-IV-TR disorder; intellectual disability per DSM-IV-TR criteria (IQ < 70 & impaired functioning); past history of head trauma with loss of consciousness; pregnancy |
| **PAFIP** | EOP | DSM-IV | Local adolescent psychiatric units or local clinical services | SCID Axis I diagnosis confirmed by an independent psychiatrist 6 months after the initial contact; written informed consent | DSM-IV criteria for (1) drug dependence (except nicotine dependence), (2) mental retardation, and when having a history of neurological disease or head injury. |
|  | HC | Comprehensive Assessment of Symptoms and History |  | Matched to patients (age, sex, laterality index, drug history, years of education); written informed consent | Current or past history of psychiatric, neurological or general medical illnesses, including substance dependence and significant loss of consciousness; presence of psychosis in first-degree relatives |
| **SRI** | EOP | KSADS-PL | Centre for Youth Bipolar Disorder | English-speaking, of any race/ethnicity, and 13-20 years of age meeting diagnostic criteria for BD (type I, II, or NOS) | 1) unable to provide informed consent, 2) existing cardiac condition (e.g. conduction abnormality or congenital heart disease), auto-immune illness, or inflammatory illness, 3) currently taking anti-inflammatory, antilipidemic, anti-hypertensive agents 4) contraindications to magnetic resonance imaging (e.g. cardiac pacemaker or other implanted device) 5) neurological or cognitive impairment, 6) infectious illness within the past 14 days, 7) substance dependence in the past 3 months |
|  | HC | KSADS-PL | Community via advertisement | English-speaking, of any race/ethnicity, and 13-20 years of age with no major psychiatric disorders and no family history of BD. | 1) unable to provide informed consent, 2) existing cardiac condition (e.g. conduction abnormality or congenital heart disease), auto-immune illness, or inflammatory illness, 3) currently taking anti-inflammatory, antilipidemic, anti-hypertensive agents 4) contraindications to magnetic resonance imaging (e.g. cardiac pacemaker or other |
| **MADRID** | EOP | K-SADS/ DSM-IV-TR | Referral from adolescent inpatient unit (Hospital General Universitario Gregorio Marañón) or local clinical services (PIENSA program) | Age between 7-18 years; onset of first psychotic positive symptom within a psychotic episode before age of 18; diagnosis of a psychotic disorder per DSM-IVTR criteria; written informed consent | Intellectual disability per DSMIV- TR criteria (IQ < 70 & impaired functioning); pervasive developmental disorder; past history of head trauma with loss of consciousness; pregnancy |
|  | HC | K-SADS/ DSM-IV-TR | Local catchment area via advertisements | Age between 7-18 years; written informed consent | Past history of psychotic illness; current diagnosis of any Axis-I DSM-IV-TR disorder; intellectual disability per DSM-IV-TR criteria (IQ < 70 & impaired functioning); past history of head trauma with loss of consciousness; pregnancy |
| **SYDNEY** | EOP | DSM-IV | Early intervention mental health services (Sydney suburbs of Camperdown or Campbelltown) | Aged 12-30; seeking help for mental health and/or substance use; willingness to participate in research study at the Brain and Mind Centre; age of onset of psychosis before 18  (Note: Age of onset of psychosis criterion applies only to this EOP VBM ENIGMA study) | History of significant neurologic disease; medical illness known to impact cognitive and brain function; intellectual and/or developmental disability (IQ < 70); current substance dependence;  insufficient English language. All subjects were asked to abstain from drug or alcohol use for 48 hours prior to testing and informed about a drug screen protocol. |
|  | HC | NA | Local area via convenience sampling and advertisements | Aged 12-20; no personal history of mental disorder  (Note: Age criterion applies only to EOP VBM ENIGMA study; HCs were drawn from a pool of HCs with a max age ~30) | History of significant neurologic disease; medical illness known to impact cognitive and brain function; intellectual and/or developmental disability (IQ < 70); current substance dependence;  insufficient English language. All subjects were asked to abstain from drug or alcohol use for 48 hours prior to testing and informed about a drug screen protocol. |
| **KCL-1** | EOP | DSM-IV | Local clinical services | DSM IV Schizophrenia, 12 - 19 years old, onset of schizophrenia before age 18, no comorbid Axis I diagnosis, no mental retardation | History of head injury, current substance misuse, any medical condition, history of hereditary disease of the nervous system |
|  | HC | NA | Local area via advertisements | Age 12 - 19, no personal history of psychiatric disorder, no family history of psychosis in first degree relatives | History of head injury, current substance misuse, any medical condition, history of hereditary disease of the nervous system |
| **FEMS** | EOP | DSM-IV | Referral from early psychosis services within the Geelong and the Southern Health sites | Psychotic disorders included: bipolar I disorder with psychotic features. schizoaffective disorder; age between 15-25 years; written informed consent; not have had a previous treated manic episode; quetiapine and lithium therapy for at least 1 month prior to randomization | Clinically relevant systemic disorder; pregnancy; sensitivity/allergy to quetiapine/lithium or their compounds; non-fluency in English; history of epilepsy; clinically relevant biochemical or haematological abnormalities; immediate risk of self-harm or risk to others; organic mental disease; IQ < 70; uncontrolled Diabetes Mellitus; use of cytochrome P450 3A4 inhibitors and/or cytochrome P450 inducers 14 days before enrolment; absolute neutrophil count of 1.5x109 per litre |
|  | HC | NA | Local catchment area via advertisements; among hospital visitors (friends of patients); via Melbourne Neuropsychiatry. Parkville. Melbourne | Matched to patients; age between 15-25 years; no history of mental illness; written informed consent | Clinically relevant systemic disorder; pregnancy; non-fluency in English; history of epilepsy; clinically relevant biochemical or haematological abnormalities; organic mental disease; IQ < 70; uncontrolled Diabetes Mellitus; use of cytochrome P450 3A4 inhibitors and/or cytochrome P450 inducers 14 days before enrollment; absolute neutrophil count of 1.5x109 per liter |

K-SADS: Kiddie Schedule for Affective Disorders and Schizophrenia, DSM: Diagnostic and Statistical Manual of Mental Disorders, SCID-5-RV: The most comprehensive version of the SCID-5, the Research Version, SCID-5-PD: The Structured Clinical Interview for DSM-5 Personality Disorders, TR: Text Revision, GAF: Global Assessment of Functioning scale, MINI: The Mini International Neuropsychiatric Interview

## Table S3 Scanner-specific image acquisition and processing details

| **Cohort** | **Scanner** | **Sequence** | **Field (Tesla)** | **Voxel size x,y,z (mm)** | **TI (ms)** | **TE (ms)** | **TR (ms)** | **Flip Angle (°)** |
| --- | --- | --- | --- | --- | --- | --- | --- | --- |
| **ROME** | Philips Achieva | 3D MPRAGE | 3.0 | 0.5,0.5,0.9; |  | 5.3 | 11 | 8 |
| **SCAPS** | GE Discovery MR750 | BRAVO-sequence | 3.0 | 1,1,1.2 |  | 3.06 | 7.90 | 12 |
| **RUND** | Siemens Sonata | 3D Spoiled Gradient Recalled (3D-SPGR) | 1.5 | 1,1,1 | 1000 | 3.93 | 2730 | 7 |
| **YTOP-1** | GE Signa HDxt | 3D T1-weighted fast spoiled gradient echo (FSPGR) | 3.0 | 1,1,1.2 | 450 | ‘minfull’ setting | 7.8 | 12 |
| **YTOP-2** | GE Discovery MR 750 | BRAVO sequence | 3.0 | 1,1,1 | 450 | 3.18 | 8.16 | 12 |
| **KCL-2** | 3T GE Signa | 3D T1-weighted | 3.0 | 1.1,1.1,1.1 |  | 2.8 | 7 | 20 |
| **OXFORD** | Siemens Sonata | 3D T1-weighted | 1.5 | 1,1,1 |  | 5.6 | 12 | 19 |
| **BARCELONA-1** | GE Genesis Signa | 3D AXIAL OBLIQ | 1.5 | 1,1,1.5 | 300 | 5.168 | 12 | 20 |
| **BARCELONA-2** | Siemens TrioTim | MPRAGE SAG IPAT ISO | 3.0 | 1,1,1 | 900 | 3.01 | 2300 | 9 |
| **PAFIP** | Philips | 3D T1-weight | 3.0 | 0.94,0.94,1 |  | 3.7 | 8.2 | 8 |
| **SRI** | Philips Achieva | 3D T1-weighted fast spoiled gradient echo imaging | 3.0 | 0.6,0.6,1.2 | 650 | 3.2 | 8.1 | 8 |
| **MADRID** | Philips Intera | 3D T1-weighted | 1.5 | 1,0.94,0.94 |  | 4.6 | 9.3 | 30 |
| **SYDNEY** | GE Discovery MR750 | MP RAGE | 3.0 | 0.9,0.9,0.9 |  | 2.78 | 7.26 | 15 |
| **KCL-1** | GE Signa | 3D T1-weighted | 1.5 | 0.86,0.86,1.5 | 300 | 4.8 | 11.2 | 18 |
| **FEMS** | Siemens TrioTim | 3T Siemens TrioTim | 3.0 | 0.9,0.9,0.9, 0.45,0.45,0.9, 1,1,1 | 900 | 2.24 | 2000 | 9 |

TI = inversion time, TR = repetition time, TE = echo time

* TR is defined as the time between inversion/preparation pulses for Siemens.

Note: In the following early onset psychosis (EOP) vs. healthy control (HC) figures, hot colours show areas where HCs have larger gray matter/white matter (GM/WM) volume than EOPs, while cold colours show areas where HCs have smaller volume than EOPs. In figures showing association between GM/WM volume and clinical covariates, hot colours show positive association while cold colours show negative association. All significant results used threshold-free cluster enhancement (TFCE) at p<0.025 FWE corrected level.

## Table S4 Regional GM volume

Regional GM volume difference analysis, controlling for age and intracranial volume (ICV), showed EOP individuals have significant reductions in GM volume in left median cingulate. No increase in GM volume was found in EOP individuals.

| Peak region | MNI coordinate | Hedges' g | Z | Cluster size (voxels) | P value  (FWE corrected) | I^2^ |
| --- | --- | --- | --- | --- | --- | --- |
| **Left median cingulate / paracingulate gyri** | **-4,-4,48** | **0.55** | **7.785** | **127295** | **0.001** | **1.41** |
| Right median cingulate / paracingulate gyri | 6,-32,42 | 0.51 | 7.447 | subcluster | 0.001 | 0.00 |
| Left anterior cingulate / paracingulate gyri | 0,20,30 | 0.51 | 7.261 | subcluster | 0.001 | 0.00 |
| Right middle temporal gyrus, BA 20 | 50,-10,-18 | 0.46 | 6.712 | subcluster | 0.001 | 0.00 |
| Right postcentral gyrus, BA 4 | 50,-8,34 | 0.45 | 6.491 | subcluster | 0.001 | 0.00 |
| Right anterior cingulate / paracingulate gyri, BA 32 | 6,48,12 | 0.44 | 6.422 | subcluster | 0.001 | 0.31 |

Mean I^2^ = 14.52

## Figure S1 Regional GM differences from 13 cohorts scanned before age 18

GM volume difference between EOP and HC, including 13 cohorts where participants were scanned before 18 years old.


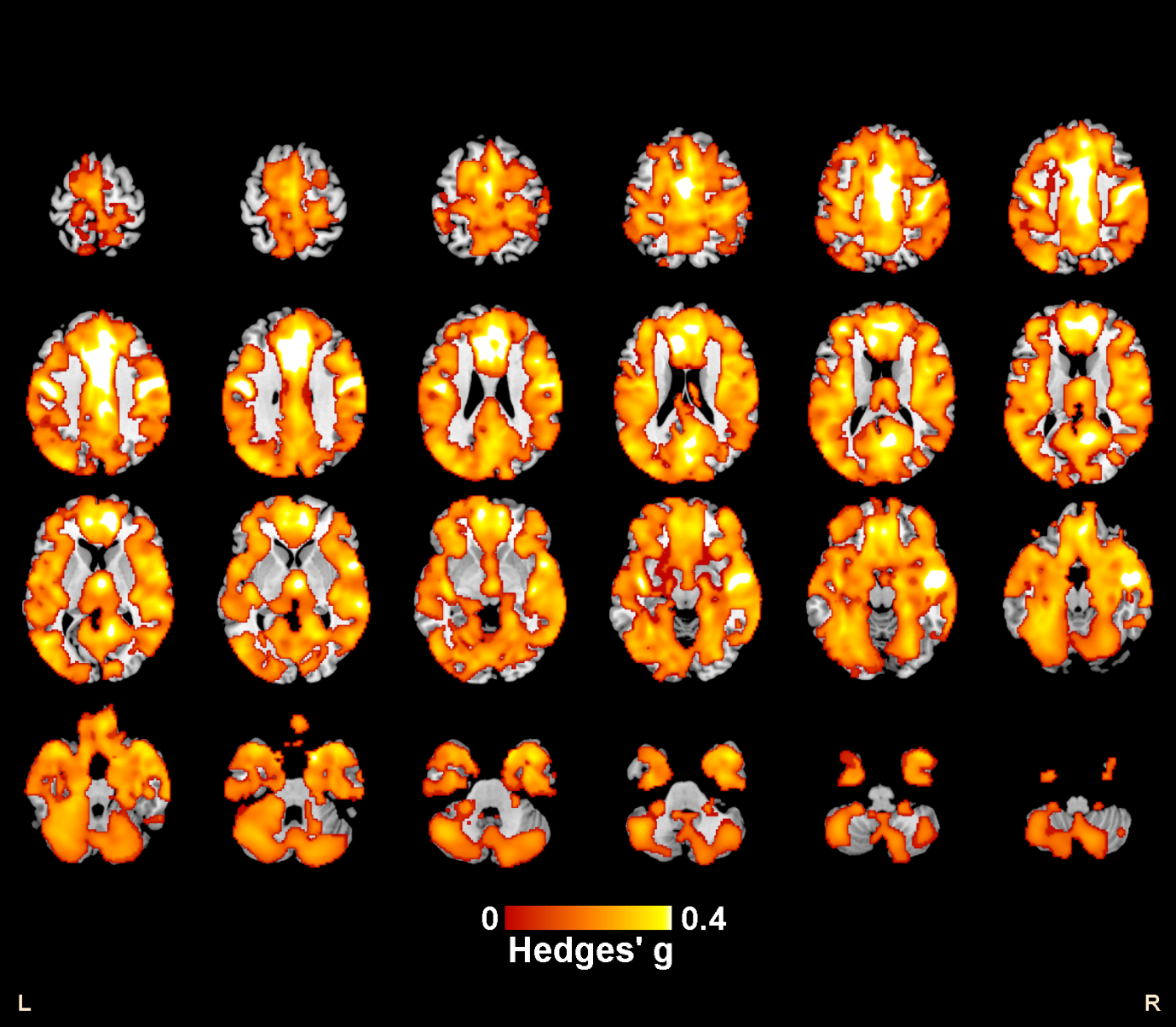


## Table S5 Regional GM differences from 13 cohorts scanned before age 18

GM volume difference between EOP and HC, including 13 cohorts where participants were scanned before 18 years old.

| Peak region | MNI coordinate | Hedges' g | Z | Cluster size (voxels) | P value  (FWE corrected) | I^2^ |
| --- | --- | --- | --- | --- | --- | --- |
| **Left median cingulate / paracingulate gyri** | **-2,16,34** | **0.53** | **7.387** | **126804** | **0.001** | **0.00** |
| Right median cingulate / paracingulate gyri | 6,-32,42 | 0.52 | 7.323 | subcluster | 0.001 | 0.00 |
| Left anterior cingulate / paracingulate gyri | -6,26,28 | 0.50 | 7.072 | subcluster | 0.001 | 0.00 |
| Right middle temporal gyrus, BA 21 | 50,-8,-18 | 0.49 | 6.829 | subcluster | 0.001 | 0.00 |
| Right postcentral gyrus, BA 4 | 50,-10,34 | 0.47 | 6.648 | subcluster | 0.001 | 0.00 |
| Right anterior cingulate / paracingulate gyri, BA 32 | 6,48,12 | 0.47 | 6.587 | subcluster | 0.001 | 0.00 |

Mean I^2^ = 16.18

## Figure S2 Regional WM volume

Regional WM volume difference analysis, controlling for age and intracranial volume (ICV), showed EOP individuals have significant reductions in WM volume in the bilateral inferior longitudinal fasciculus, right superior longitudinal fasciculus, and other regions. No increase in WM volume was found in EOP individuals.


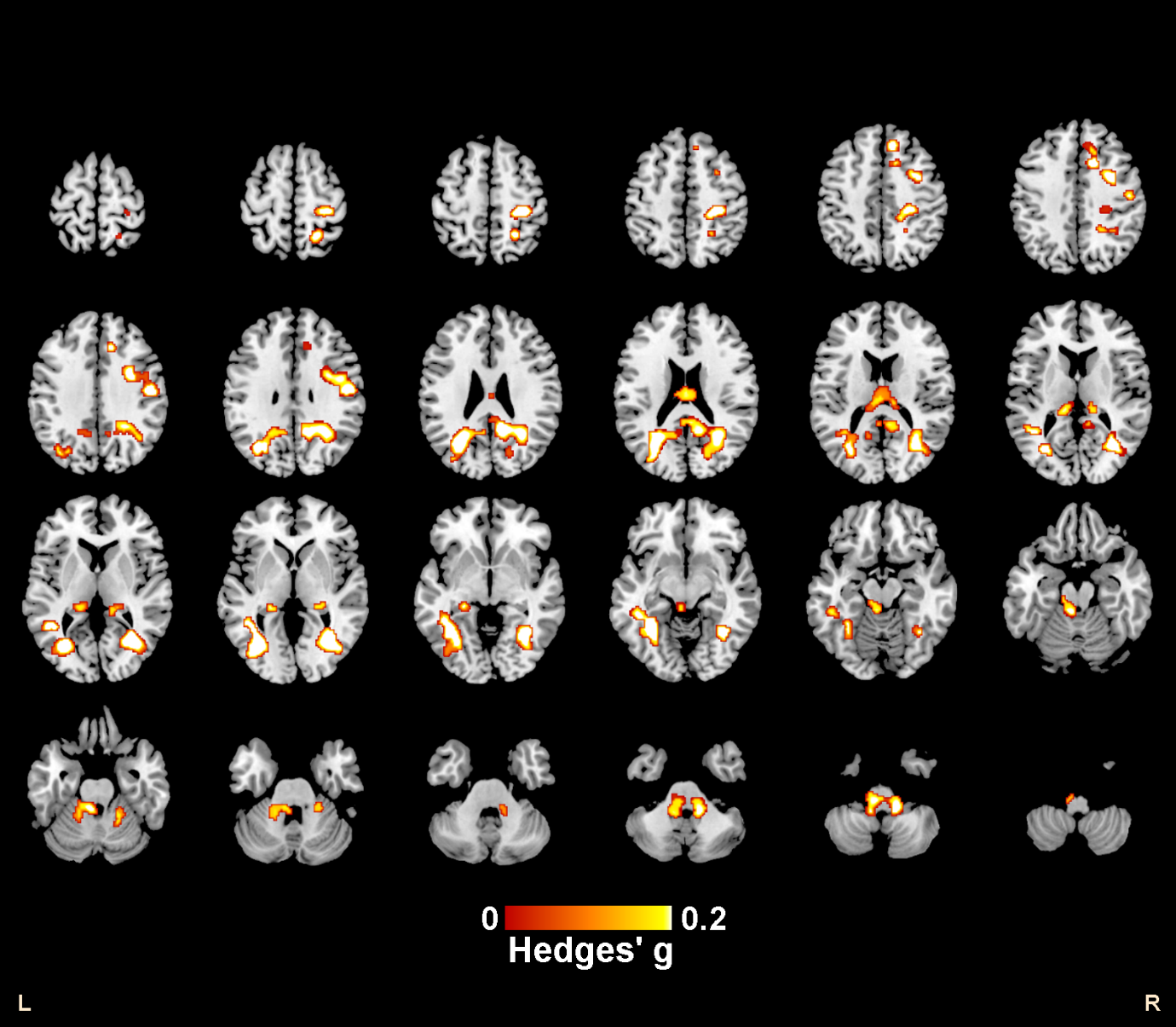


## Table S6 Regional WM volume

| Peak region | MNI coordinate | Hedges' g | Z | Cluster size (voxels) | P value  (FWE corrected) | I^2^ |
| --- | --- | --- | --- | --- | --- | --- |
| Right inferior network, inferior longitudinal fasciculus | 32,-64,2 | 0.33 | 4.839 | 2129 | 0.001 | 0.00 |
| Left inferior network, inferior longitudinal fasciculus | -34,-70,2 | 0.32 | 4.696 | 1924 | 0.001 | 0.00 |
| Right superior longitudinal fasciculus III | 50,-14,34 | 0.29 | 4.258 | 573 | 0.003 | 0.00 |
| Left anterior thalamic projections | -2,-20,16 | 0.22 | 3.204 | 437 | 0.017 | 0.00 |
| Left cerebellum | -8,-38,-22 | 0.22 | 3.219 | 319 | 0.017 | 0.00 |
| Corpus callosum | 10,32,46 | 0.24 | 3.431 | 121 | 0.017 | 0.00 |
| Right cerebellum | 22,-38,-28 | 0.20 | 2.899 | 60 | 0.023 | 0.00 |

Mean I^2^ = 5.52

## Figure S3 Global WM

Global WM showed no statistically significant result.


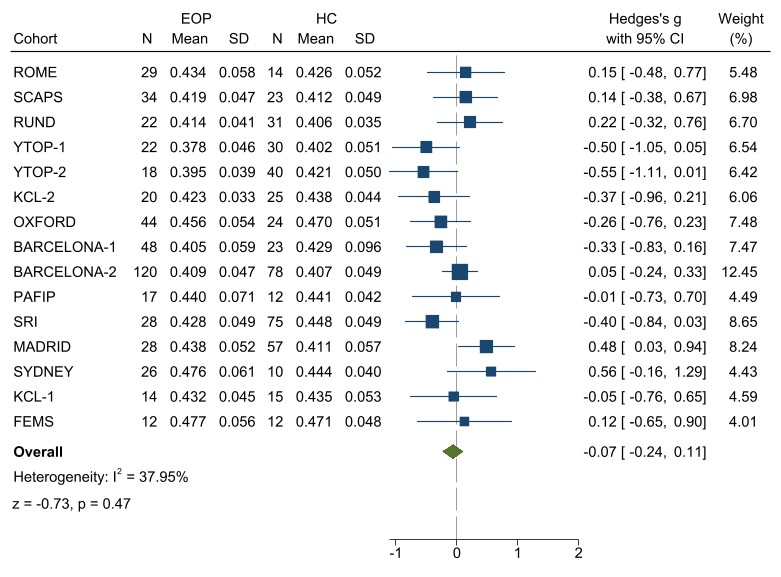


## Table S7 Regional GM volume differences associated with age of onset

| Peak region | MNI coordinate | Hedges' g | Z | Cluster size (voxels) | P value  (FWE corrected) | I^2^ |
| --- | --- | --- | --- | --- | --- | --- |
| Right cerebellum, hemispheric lobule VI, BA 37 | 40,-42,-30 | -0.20 | -4.022 | 654 | 0.002 | 0.00 |
| Left cerebellum | -6,-48,-60 | -0.17 | -3.345 | 161 | 0.015 | 0.00 |
| Left inferior parietal gyri | -26,-44,48 | -0.24 | -4.815 | 123 | 0.002 | 0.00 |
| Right thalamus | 2,-20,6 | -0.17 | -3.374 | 58 | 0.022 | 0.00 |

* Age of onset is available from 439 individuals from 14 cohorts

Mean I^2^ = 8.71

## Figure S4 Regional WM volume differences associated with age of onset

Higher volume in middle cerebellar peduncles was associated with younger age of onset.


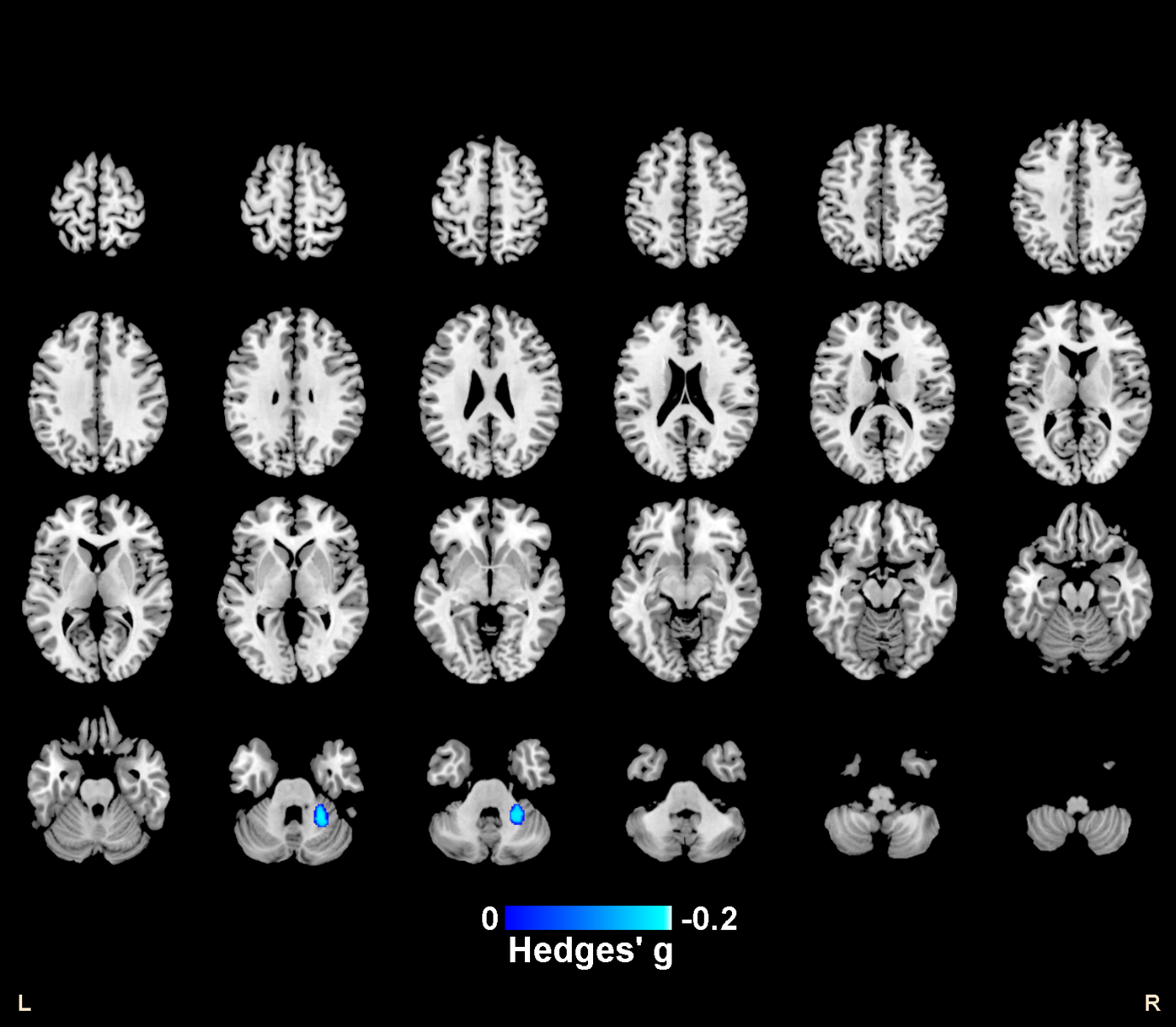


* Age of onset is available from 439 individuals from 14 cohorts

## Table S8 Regional WM volume differences associated with age of onset

| Peak region | MNI coordinate | Hedges' g | Z | Cluster size (voxels) | P value (FWE corrected) | I^2^ |
| --- | --- | --- | --- | --- | --- | --- |
| Middle cerebellar peduncles | 26,-46,-32 | -0.18 | -3.673 | 194 | 0.012 | 0.00 |

* Age of onset is available from 439 individuals from 14 cohorts

Mean I^2^ = 8.71

## Table S9 Regional GM differences associated with CPZ equivalent dose

Widespread lower volume in multiple regions was associated with a higher chlorpromazine (CPZ) equivalent dose (Peak region left inferior frontal gyrus, Hedges' g=-0.24, p =0.001). There was no positive association between GM and CPZ.

| Peak region | MNI coordinate | Hedges' g | Z | Cluster size (voxels) | P value (FWE corrected) | I^2^ |
| --- | --- | --- | --- | --- | --- | --- |
| Left inferior frontal gyrus, orbital part, BA 47 | -40,36,-18 | -0.24 | -4.424 | 9878 | 0.001 | 0.00 |
| Right insula | 42,-8,-2 | -0.15 | -2.795 | 1042 | 0.017 | 0.00 |
| Right precentral gyrus, BA 6 | 52,-4,42 | -0.18 | -3.224 | 178 | 0.016 | 0.00 |
| Right superior frontal gyrus, dorsolateral | 26,30,48 | -0.18 | -3.356 | 158 | 0.016 | 0.00 |

*CPZ data is available from 342 EOP individuals from 10 cohorts

Mean I^2^ = 5.44

## Figure S5 Regional GM differences associated with duration of illness

Higher GM volume in the right precentral gyrus, fusiform gyrus and left cerebellum was associated with longer duration of illness. There was no negative association between GM and duration of illness.


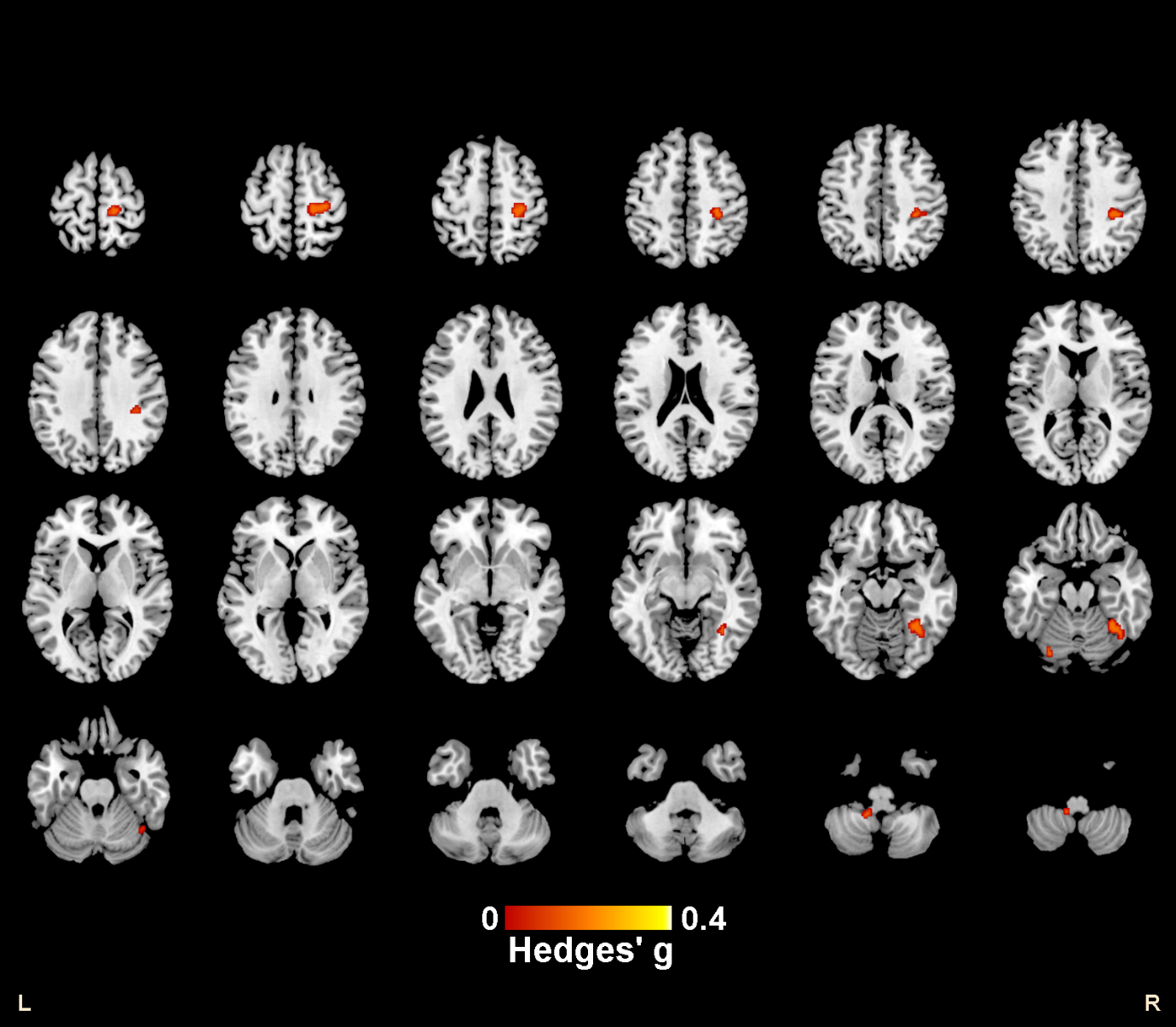


*Duration of illness data is available from 427 EOP individuals from 13 cohorts

## Table S10 Regional GM differences associated with duration of illness

| Peak region | MNI coordinate | Hedges' g | Z | Cluster size (voxels) | P value (FWE corrected) | I^2^ |
| --- | --- | --- | --- | --- | --- | --- |
| Right precentral gyrus, BA 4 | 12,-30,70 | 0.18 | 3.481 | 334 | 0.0190 | 0.00 |
| Right fusiform gyrus, BA 37 | 30,-50,-18 | 0.19 | 3.715 | 144 | 0.0190 | 0.00 |
| Left cerebellum | -6,-50,-62 | 0.17 | 3.244 | 37 | 0.022 | 0.00 |

*Duration of illness data is available from 427 EOP individuals from 13 cohorts

Mean I^2^ = 8.92

## Figure S6 Regional GM differences associated with IQ

Higher GM volume was correlated with higher IQ (Peak region: right temporal pole, Hedges' g=0.35, p =0.001). There was no negative association between GM and IQ.


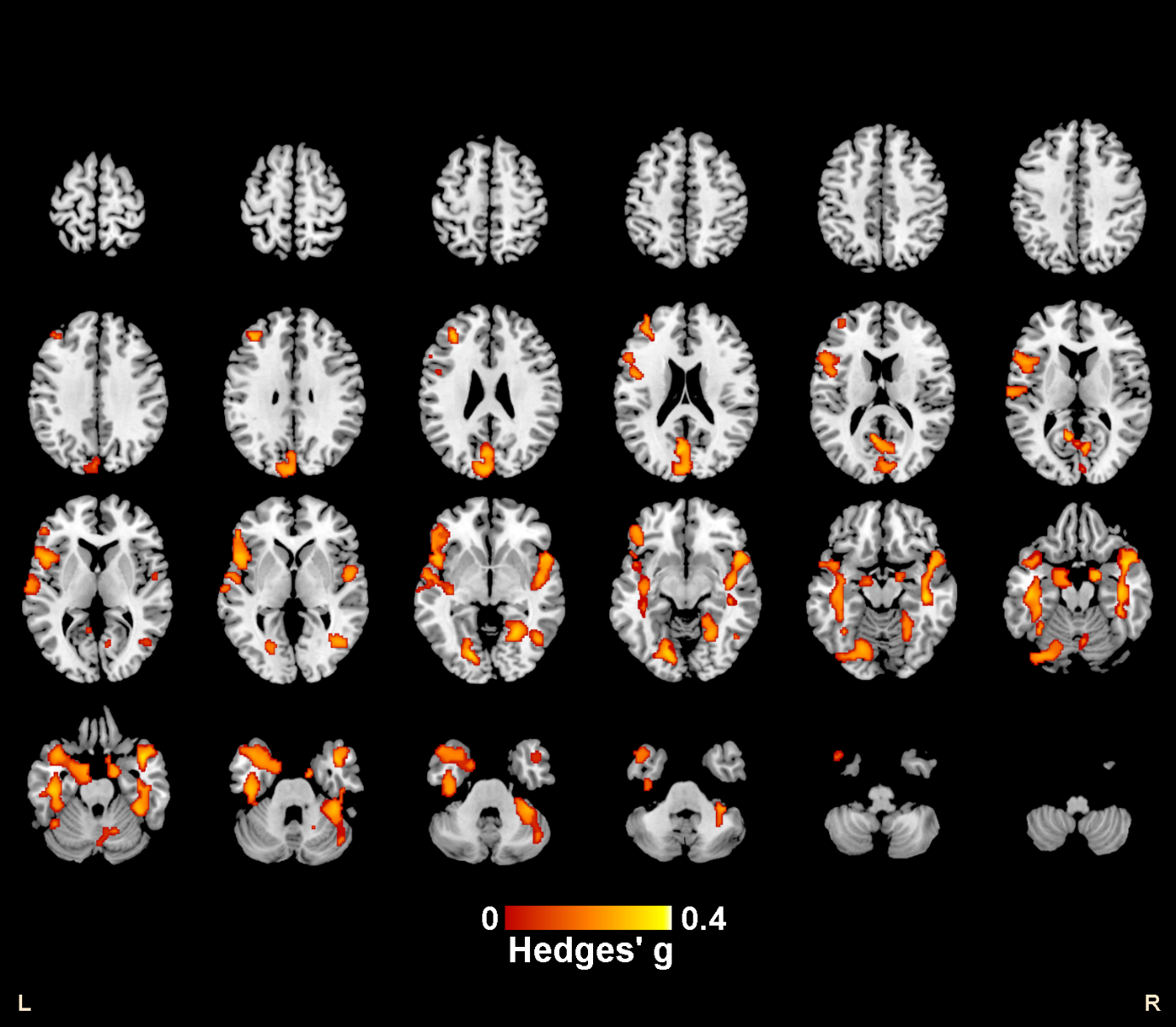


* IQ data is available from 141 EOP individuals from 6 cohorts

## Table S11 Regional GM differences associated with IQ

| Peak region | MNI coordinate | Hedges' g | Z | Cluster size (voxels) | P value (FWE corrected) | I^2^ |
| --- | --- | --- | --- | --- | --- | --- |
| Left temporal pole, superior temporal gyrus, BA 38 | -38,12,-26 | 0.31 | 3.591 | 3671 | 0.002 | 0.00 |
| Right temporal pole, superior temporal gyrus, BA 38 | 42,-24,-14 | 0.35 | 3.925 | 1908 | 0.001 | 0.00 |
| Left cuneus cortex | -6,-88,24 | 0.30 | 3.396 | 918 | 0.002 | 0.00 |
| Left fusiform gyrus | -18,-78,-6 | 0.34 | 3.921 | 686 | 0.002 | 0.00 |
| Right lingual gyrus | 24,-58,-4 | 0.32 | 3.678 | 593 | 0.002 | 0.00 |
| Left inferior frontal gyrus, triangular part, BA 46 | -36,36,26 | 0.31 | 3.557 | 187 | 0.007 | 0.00 |
| Right parahippocampal gyrus, BA 34 | 16,-2,-18 | 0.28 | 3.244 | 157 | 0.013 | 0.00 |
| Right cerebellum | 16,-58,-28 | 0.21 | 2.383 | 58 | 0.025 | 0.00 |

* IQ data is available from 141 EOP individuals from 6 cohorts

Mean I^2^ = 8.91

## Figure S7 Regional GM differences from 6 cohorts without AFP

This analysis was restricted to 147 EOP and 169 HC from 6 cohorts that did not have AFP individuals.


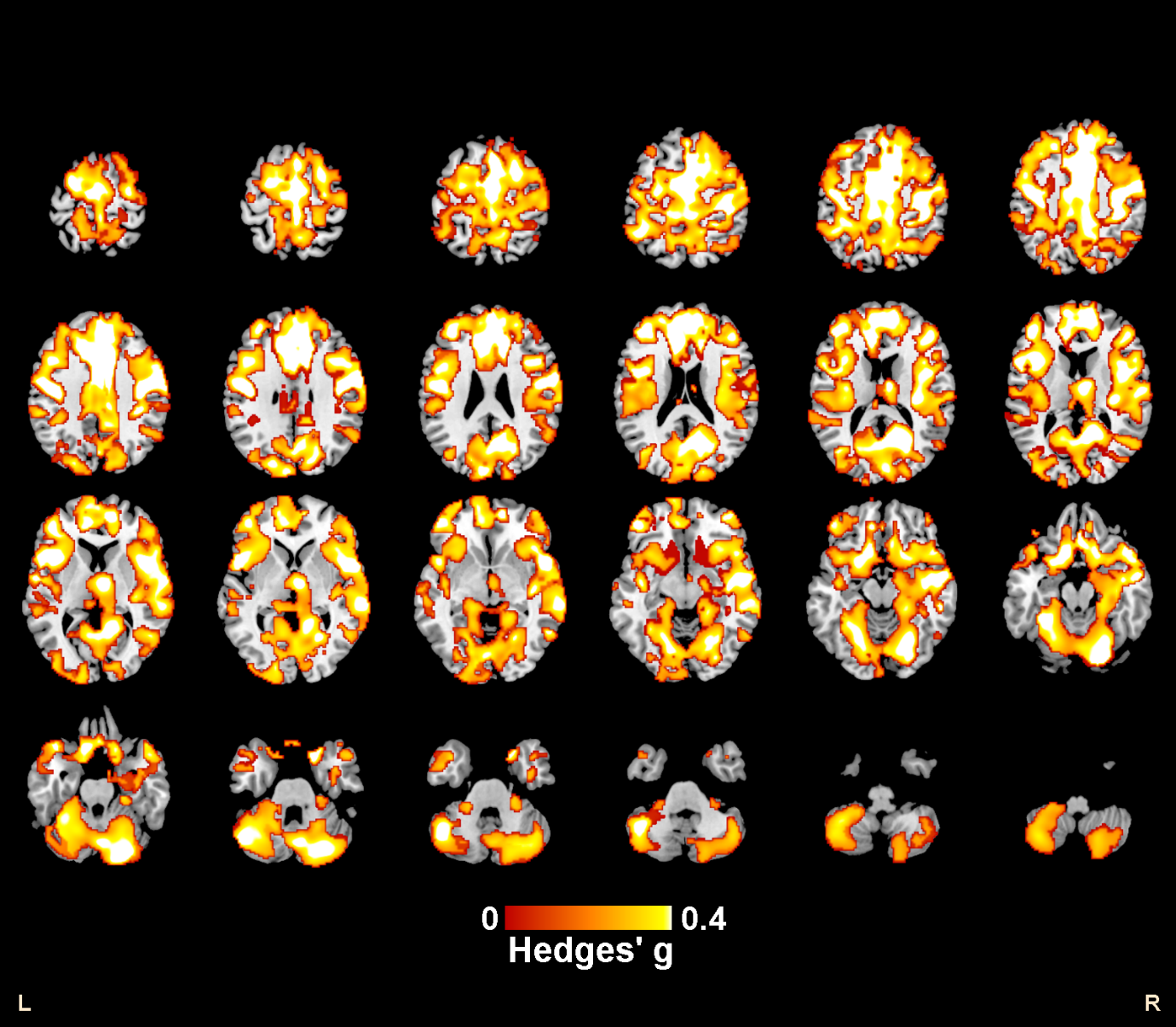


## Table S12 Regional GM differences from 6 cohorts without AFP

This analysis was restricted to 147 EOP and 169 HC from 6 cohorts that did not have AFP individuals.

| Peak region | MNI coordinate | Hedges' g | Z | Cluster size (voxels) | P value  (FWE corrected) | I^2^ |
| --- | --- | --- | --- | --- | --- | --- |
| **Left median cingulate / paracingulate gyri** | **-6,-4,48** | **0.72** | **6.008** | **80279** | **0.001** | **0.00** |
| Right postcentral gyrus, BA 4 | 56,-4,34 | 0.64 | 5.386 | subcluster | 0.001 | 0.00 |
| Right precentral gyrus, BA 4 | 50,-8,36 | 0.62 | 5.227 | subcluster | 0.001 | 0.00 |
| Right insula, BA 48 | 44,8,4 | 0.59 | 4.970 | subcluster | 0.001 | 0.00 |
| Left postcentral gyrus, BA 4 | -48,-8,32 | 0.59 | 4.918 | subcluster | 0.001 | 0.00 |
| Left anterior cingulate / paracingulate gyri | -6,26,30 | 0.59 | 4.877 | subcluster | 0.001 | 4.04 |

Mean I^2^ = 21.53

# Regional GM differences analysis between EOP and HC controlling for different covariates

All analyses controlled for age and ICV used subjects from 15 cohorts, with 482 EOP individuals and 469 HCs. All analyses controlled for age, ICV and sex used subjects from 14 cohorts with 460 EOP individuals and 444 HCs, because KCL-2 only has male subjects.

## Figure S8 GM controlling for Age, ICV and sex


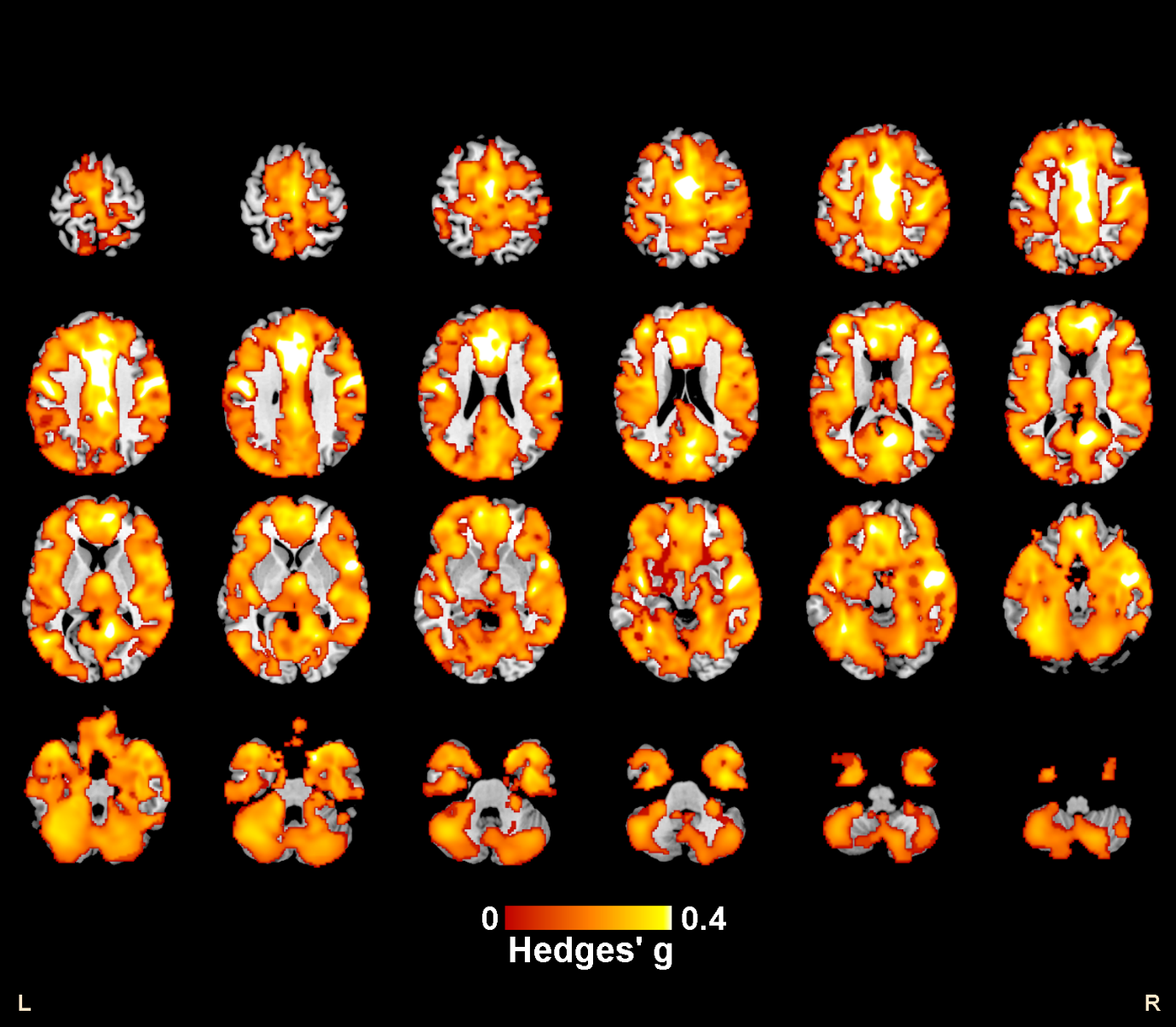


## Table S13 GM controlling for Age, ICV and sex

| Peak region | MNI coordinate | Hedges' g | Z | Cluster size (voxels) | P value (FWE corrected) | I^2^ |
| --- | --- | --- | --- | --- | --- | --- |
| **Left median cingulate / paracingulate gyri** | **-2,-4,48** | **0.60** | **8.371** | **126782** | **0.001** | **0.00** |
| Right median cingulate / paracingulate gyri | 6,-32,42 |  | 7.365 | subcluster | 0.001 | 0.00 |
| Right insula, BA 48 | 50,6,-4 |  | 6.471 | subcluster | 0.001 | 1.58 |
| Right postcentral gyrus, BA 4 | 58,-4,32 |  | 6.376 | subcluster | 0.001 | 0.00 |
| Right anterior cingulate / paracingulate gyri, BA 32 | 4,48,10 |  | 6.267 | subcluster | 0.001 | 0.00 |
| Left anterior cingulate / paracingulate gyri | 0,46,12 |  | 6.184 | subcluster | 0.001 | 0.00 |

Mean I^2^ = 16.29

## Figure S9 GM controlling for Age and total GM volume

Comparing with healthy controls, EOP individuals had reduced GM volume peaking in left median cingulate (Hedges’ g =0.44, p =0.001), and increased left striatum (Hedges’ g =0.-42, p=0.001).


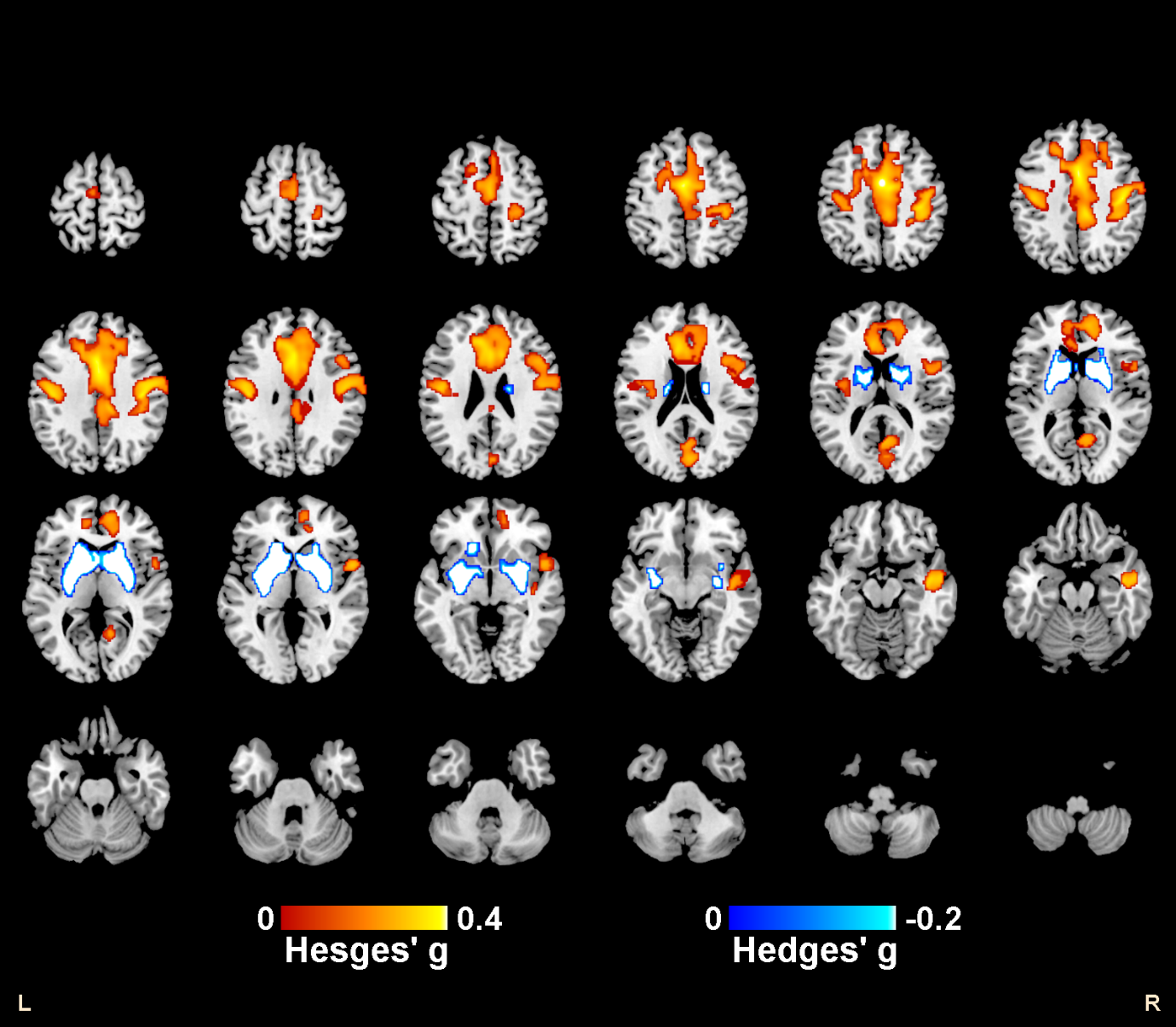


## Table S14 GM controlling for Age and total GM volume

| Peak region | MNI coordinate | Hedges' g | Z | Cluster size (voxels) | P value (FWE corrected) | I^2^ |
| --- | --- | --- | --- | --- | --- | --- |
| Left median cingulate / paracingulate gyri | -2,-4,48 | 0.44 | 6.438 | 8247 | 0.001 | 0.00 |
| Right postcentral gyrus | 42,-18,38 | 0.39 | 5.711 | 2222 | 0.001 | 0.00 |
| Right cuneus cortex, BA 18 | 4,-78,20 | 0.30 | 4.313 | 424 | 0.011 | 0.00 |
| Right middle temporal gyrus | 44,-10,-18 | 0.35 | 4.974 | 365 | 0.003 | 0.00 |
| Right striatum | 16,4,6 | -0.42 | -6.097 | 3694 | 0.001 | 0.00 |

Mean I^2^ = 9.83

## Figure S10 GM controlling for ICV only


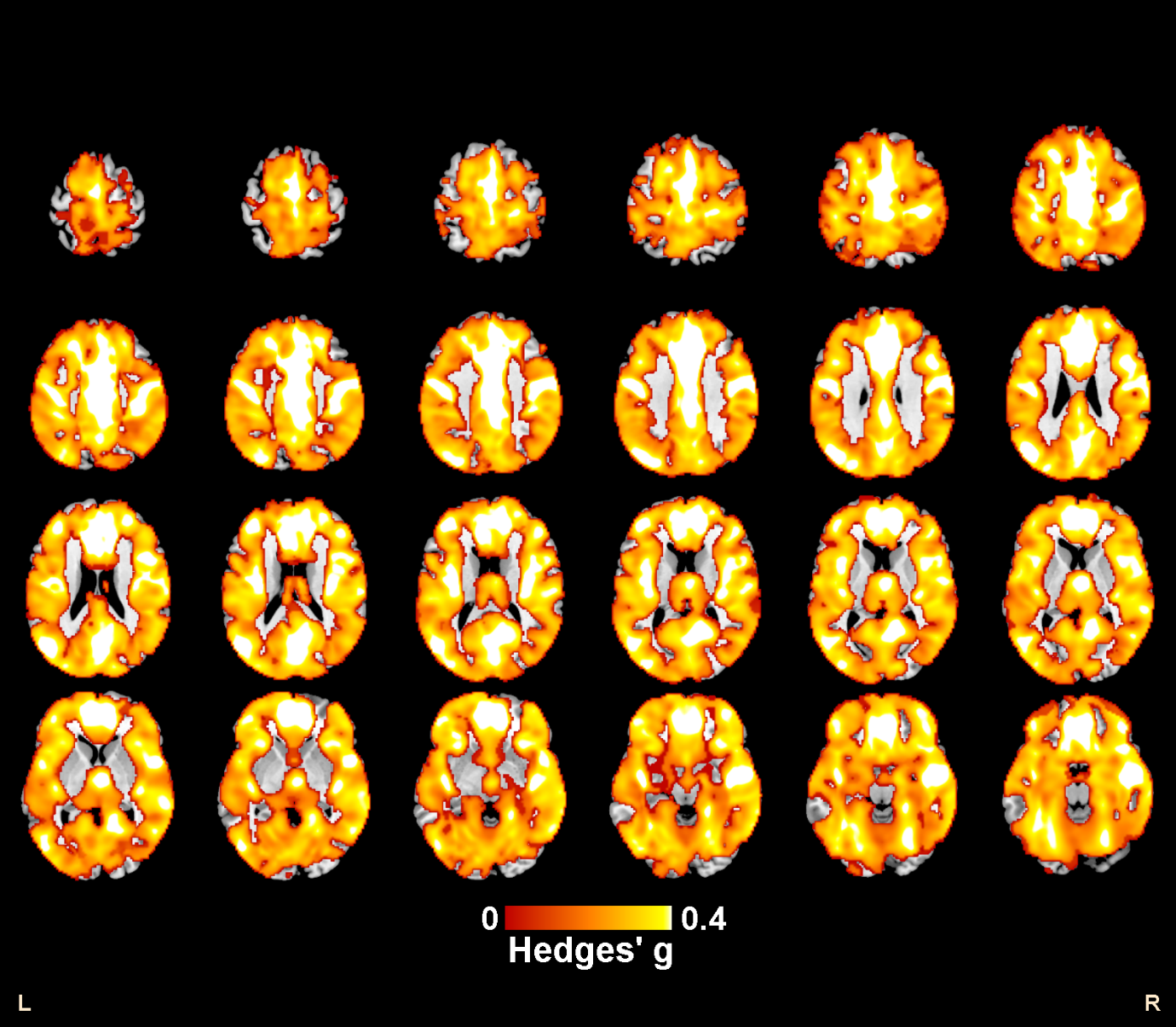


## Table S15 GM controlling for ICV only

| Peak region | MNI coordinate | Hedges' g | Z | Cluster size (voxels) | P value (FWE corrected) | I^2^ |
| --- | --- | --- | --- | --- | --- | --- |
| This is a large cluster compressing right precentral gyrus, left cuneus cortex, right inferior frontal gyrus, right median cingulate / paracingulate gyri | 42,-18,34 | 0.54 | 7.799 | 160719 | 0.001 | 0.00 |
| Right precentral gyrus, BA 4 | 44,-10,40 | 0.52 | 7.335 | subcluster | 0.001 | 2.38 |
| Left cuneus cortex, BA 18 | 2,-76,20 | 0.50 | 7.272 | subcluster | 0.001 | 0.00 |
| Right calcarine fissure / surrounding cortex, BA 17 | 6,-62,10 | 0.50 | 7.229 | subcluster | 0.001 | 0.00 |
| Right postcentral gyrus, BA 43 | 60,-6,30 | 0.48 | 6.894 | subcluster | 0.001 | 0.00 |
| Right inferior frontal gyrus, triangular part, BA 48 | 44,12,24 | 0.50 | 6.847 | subcluster | 0.001 | 6.16 |

Mean I^2^ = 28.68

## Figure S11 GM using Proportional scaling, controlling for age and ICV


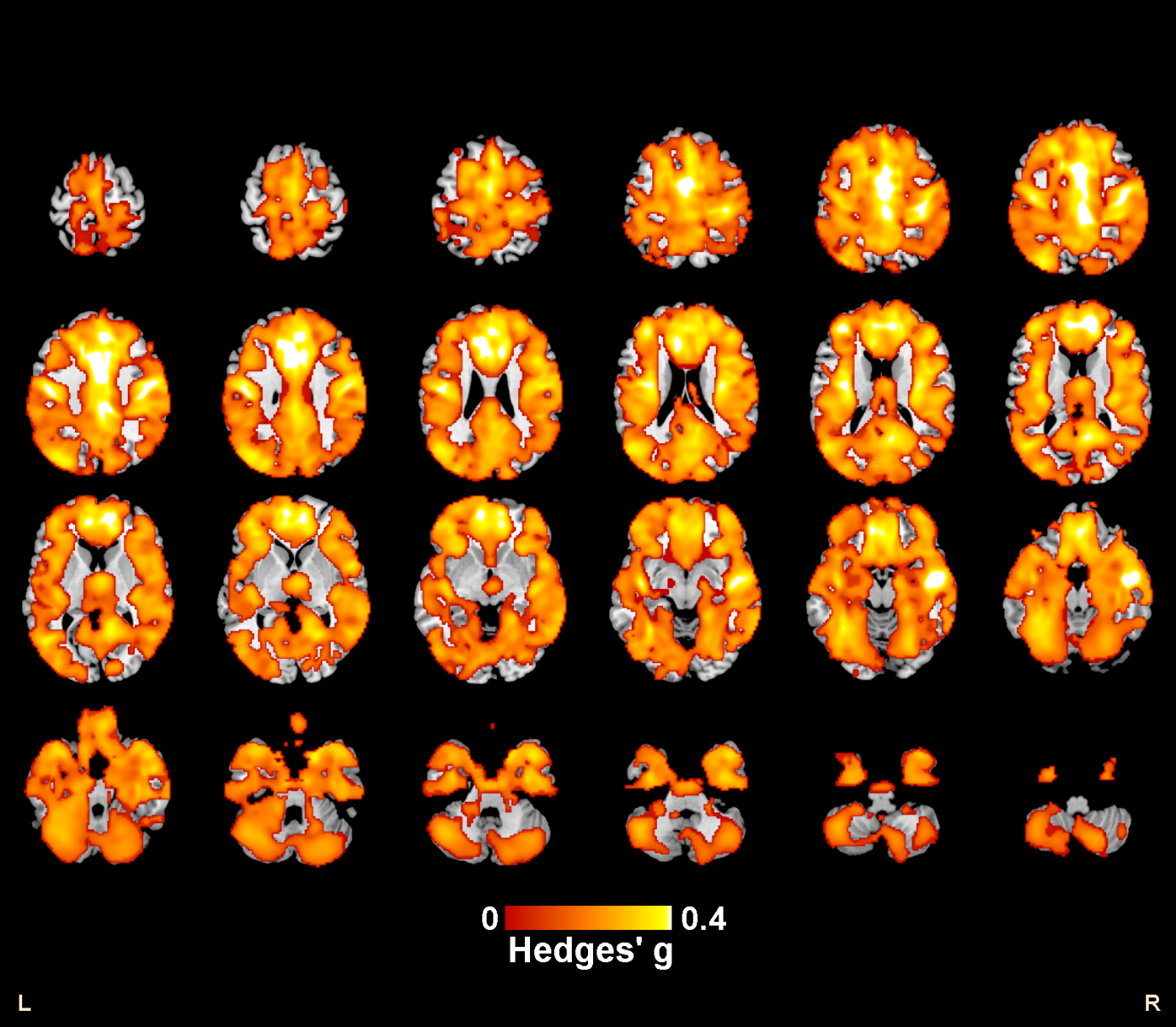


## Table S16 GM using Proportional scaling, controlling for age and ICV

| Peak region | MNI coordinate | Hedges' g | Z | Cluster size (voxels) | P value (FWE corrected) | I^2^ |
| --- | --- | --- | --- | --- | --- | --- |
| **Left median cingulate / paracingulate gyri** | **-4,-4,48** | **0.51** | **7.364** | **134634** | **0.001** | **0.00** |
| Right middle temporal gyrus, BA 20 | 50,-10,-18 | 0.48 | 6.991 | subcluster | 0.001 | 0.00 |
| Left anterior cingulate / paracingulate gyri, BA 24 | -6,26,30 | 0.47 | 6.749 | subcluster | 0.001 | 0.00 |
| Left supplementary motor area | -2,-12,48 | 0.46 | 6.613 | subcluster | 0.001 | 0.00 |
| Right anterior cingulate / paracingulate gyri, BA 32 | 2,48,10 | 0.44 | 6.334 | subcluster | 0.001 | 0.00 |
| Right median cingulate / paracingulate gyri | 8,-14,42 | 0.41 | 6.001 | subcluster | 0.001 | 0.00 |

Mean I^2^ = 16.77

## Figure S12 GM controlling for age, age squared, ICV, sex

Controlling for age squared models the effect a differing age, rather than assuming the effect is linear for all ages.


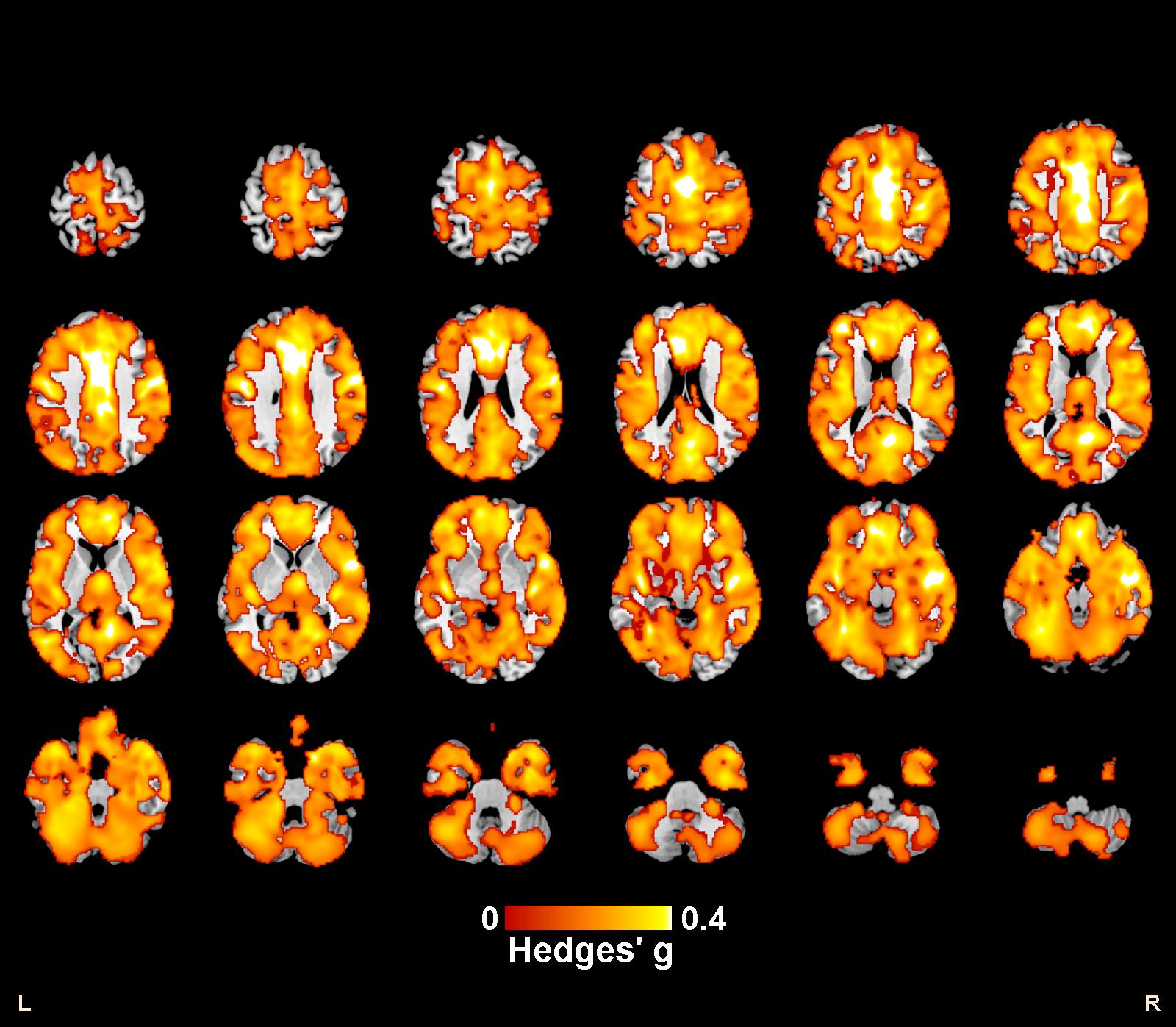


## Table S17 GM controlling for age, age squared, ICV, sex

| Peak region | MNI coordinate | Hedges' g | Z | Cluster size (voxels) | P value (FWE corrected) | I^2^ |
| --- | --- | --- | --- | --- | --- | --- |
| **Left median cingulate / paracingulate gyri** | **0,-4,46** | **0.59** | **8.228** | **131426** | **0.001** | **0.00** |
| Right median cingulate / paracingulate gyri | 6,-32,42 | 0.51 | 7.261 | subcluster | 0.001 | 0.00 |
| Left anterior cingulate / paracingulate gyri, BA 32 | -10,30,26 | 0.48 | 6.789 | subcluster | 0.001 | 0.00 |
| Right rolandic operculum, BA 48 | 52,8,-2 | 0.46 | 6.529 | subcluster | 0.001 | 0.00 |
| Right calcarine fissure / surrounding cortex, BA 17 | 8,-60,12 | 0.46 | 6.464 | subcluster | 0.001 | 0.00 |
| Right temporal pole, superior temporal gyrus, BA 48 | 56,10,-2 | 0.44 | 6.284 | subcluster | 0.001 | 0.00 |

Mean I^2^ = 15.03

## Figure S13 GM not controlling for any covariate


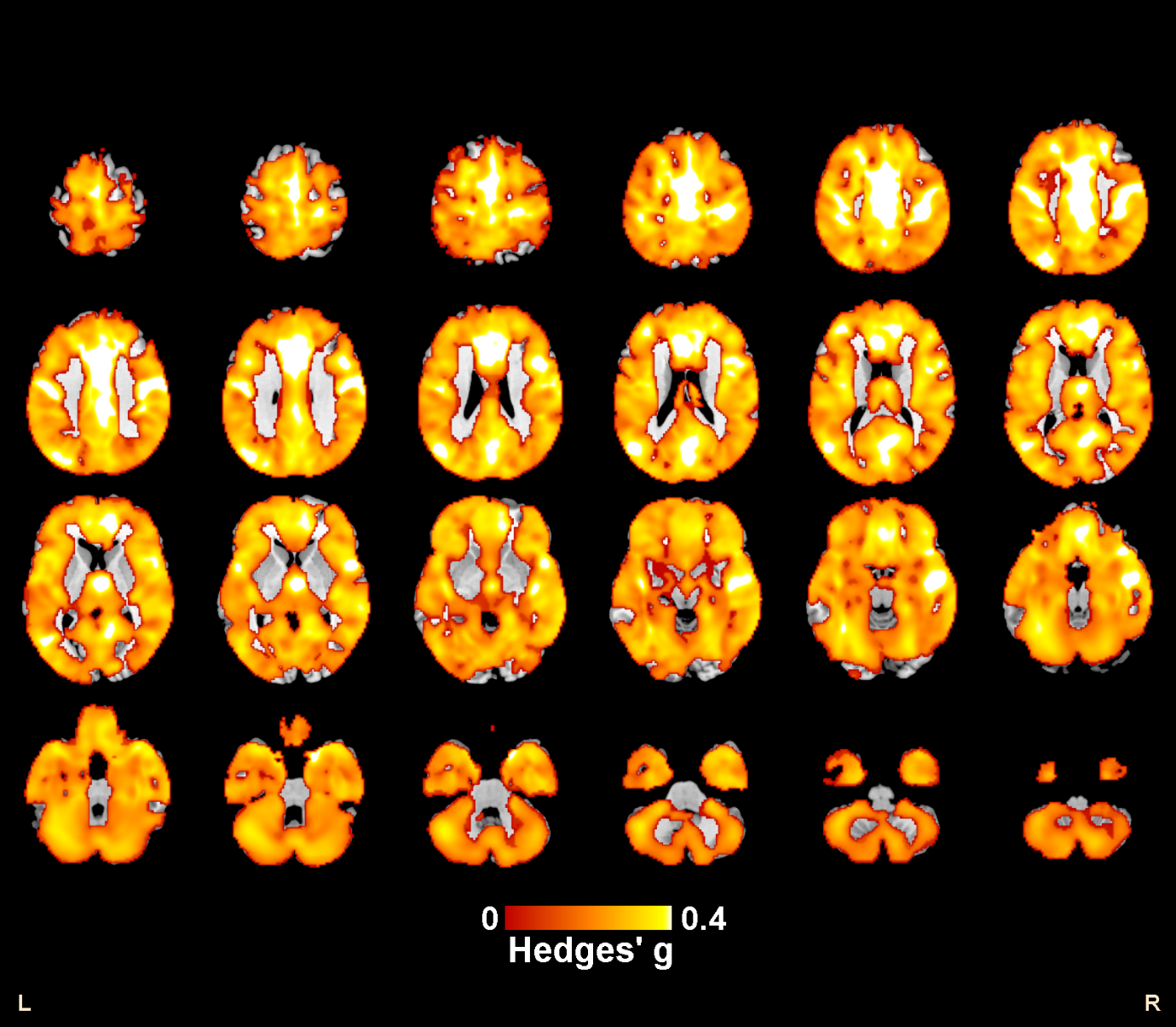


## Table S18 GM not controlling for any covariate

| Peak region | MNI coordinate | Hedges' g | Z | Cluster size (voxels) | P value (FWE corrected) | I^2^ |
| --- | --- | --- | --- | --- | --- | --- |
| **Left median cingulate / paracingulate gyri, BA 24** | **-2,16,32** | **0.58** | **8.056** | **170475** | **<0.001** | **0.00** |
| Left supplementary motor area | -2,-10,50 | 0.56 | 8.014 | subcluster | 0.001 | 0.00 |
| Right thalamus | 6,-8,2 | 0.47 | 6.874 | subcluster | 0.001 | 0.00 |
| Right inferior frontal gyrus, triangular part, BA 48 | 44,12,24 | 0.48 | 6.751 | subcluster | 0.001 | 3.62 |
| Left cuneus cortex | 2,-78,20 | 0.46 | 6.738 | subcluster | 0.001 | 0.00 |
| Right median cingulate / paracingulate gyri | 10,-16,44 | 0.47 | 6.720 | subcluster | 0.001 | 1.59 |

Mean I^2^ = 21.24

# Regional WM differences analysis between EOP and HC controlling for different covariates

## Figure S14 WM controlling for Age, ICV, sex


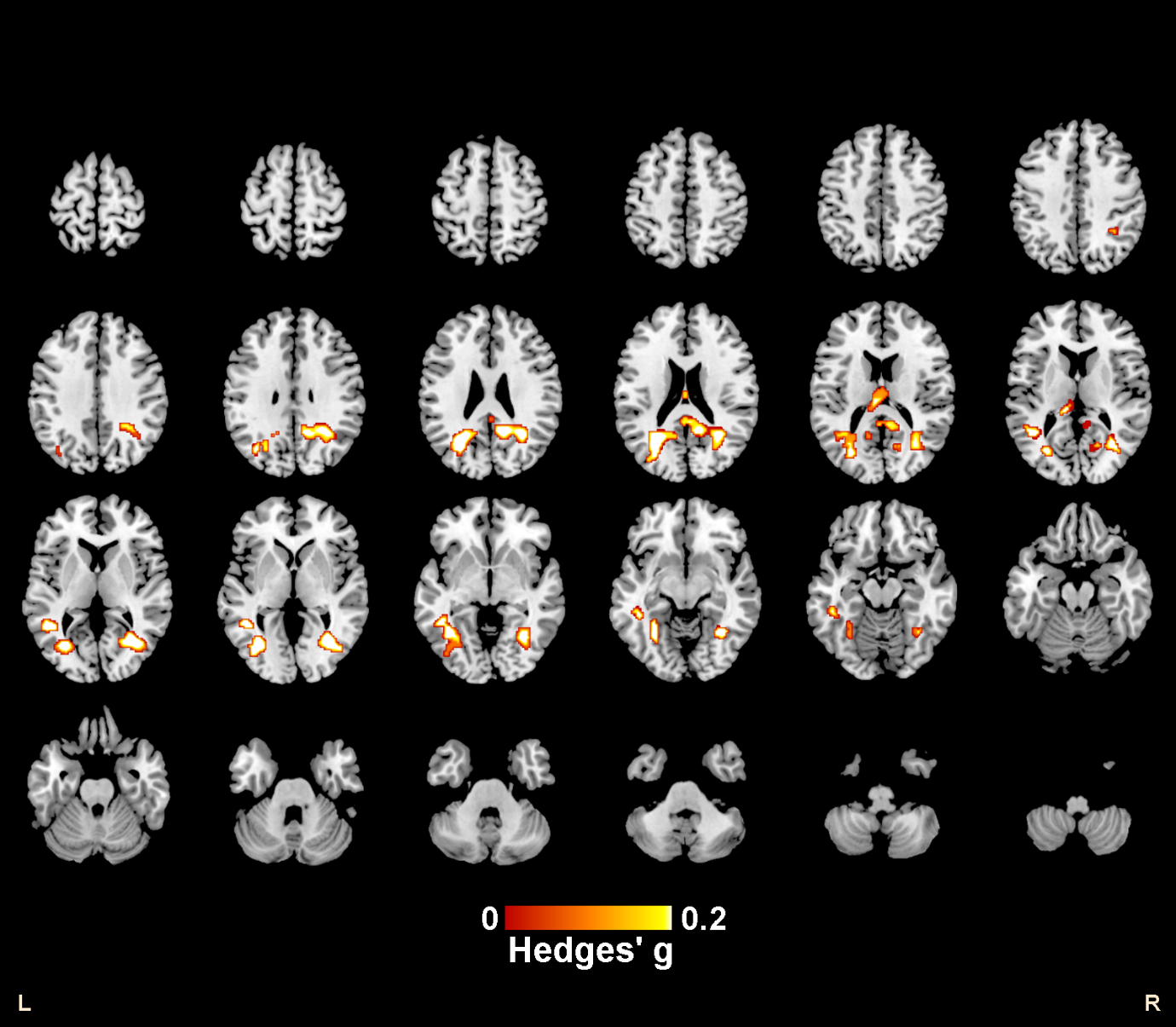


## Table S19 WM controlling for Age, ICV, sex

| Peak region | MNI coordinate | Hedges' g | Z | Cluster size (voxels) | P value (FWE corrected) | I^2^ |
| --- | --- | --- | --- | --- | --- | --- |
| Right inferior network, inferior longitudinal fasciculus | 32,-64,2 | 0.29 | 4.129 | 1387 | 0.001 | 0.00 |
| Corpus callosum | -28,-62,24 | 0.29 | 4.095 | 1340 | 0.001 | 0.00 |
| Left anterior thalamic projections | -4,-20,16 | 0.22 | 3.199 | 126 | 0.021 | 0.00 |

Mean I^2^ = 5.64

## Figure S15 WM controlling for Age and total WM volume


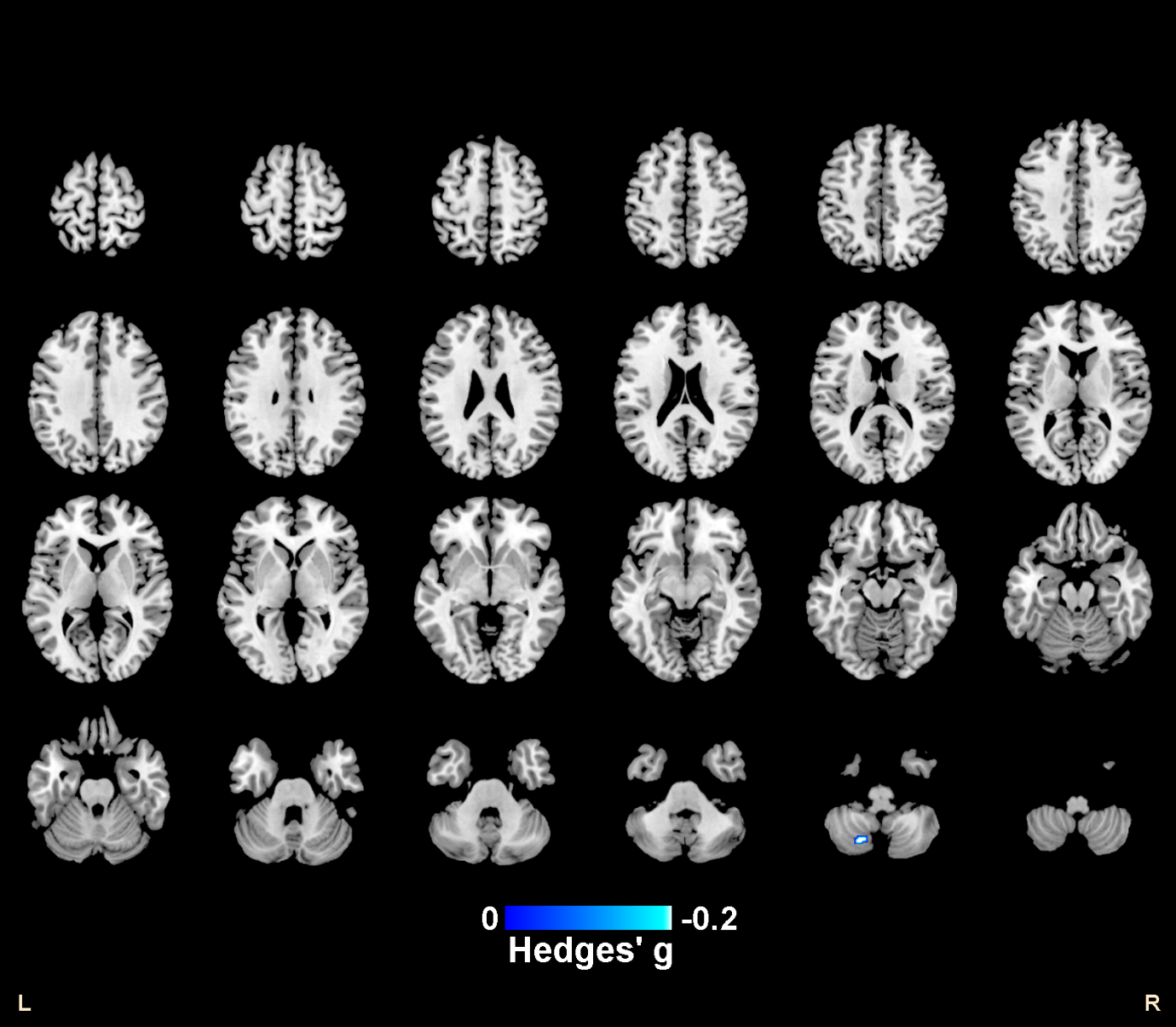


## Table S20 WM controlling for Age and total WM volume

| Peak region | MNI coordinate | Hedges' g | Z | Cluster size (voxels) | P value (FWE corrected) | I^2^ |
| --- | --- | --- | --- | --- | --- | --- |
| Left cerebellum, hemispheric lobule VIII | -20,-66,-48 | -0.41 | -5.902 | 25 | 0.011 | 0.00 |

Mean I^2^ = 4.56

## Figure S16 WM controlling for ICV


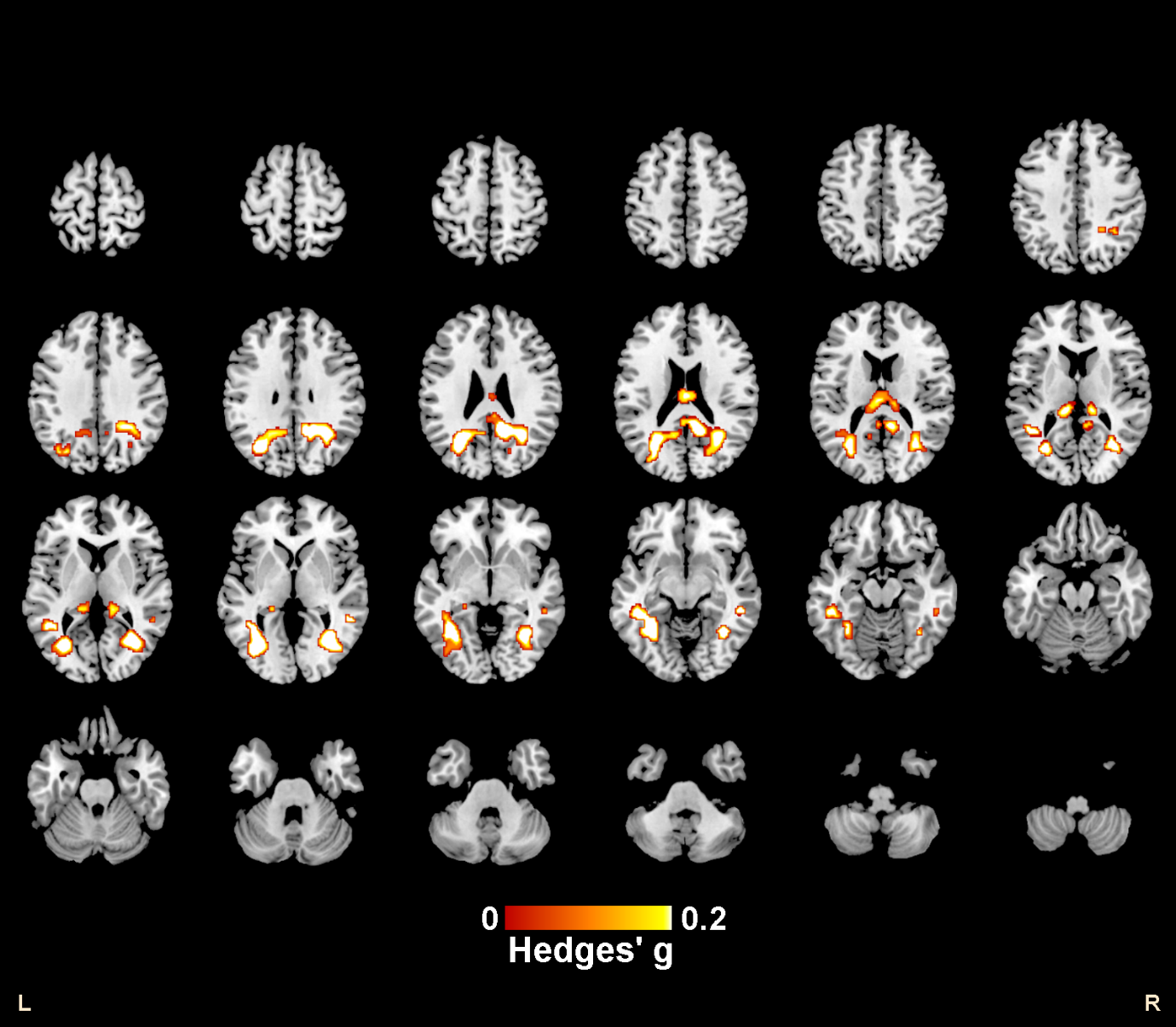


## Table S21 WM controlling for ICV

| Peak region | MNI coordinate | Hedges' g | Z | Cluster size (voxels) | P value (FWE corrected) | I^2^ |
| --- | --- | --- | --- | --- | --- | --- |
| Left inferior network, inferior longitudinal fasciculus | -36,-70,0 | 0.31 | 4.512 | 1875 | 0.001 | 0.00 |
| Right inferior network, inferior longitudinal fasciculus | 32,-64,2 | 0.31 | 4.539 | 1778 | 0.001 | 0.00 |
| Left anterior thalamic projections | -2,-20,16 | 0.23 | 3.343 | 400 | 0.010 | 0.00 |
| Corpus callosum | 12,-30,12 | 0.21 | 3.863 | 38 | 0.019 | 0.00 |
| Right arcuate network, posterior segment | 52,-44,2 | 0.26 | 3.838 | 22 | 0.021 | 0.00 |

Mean I^2^ = 6.56

## Figure S17 WM using Proportional scaling, controlling for age and ICV


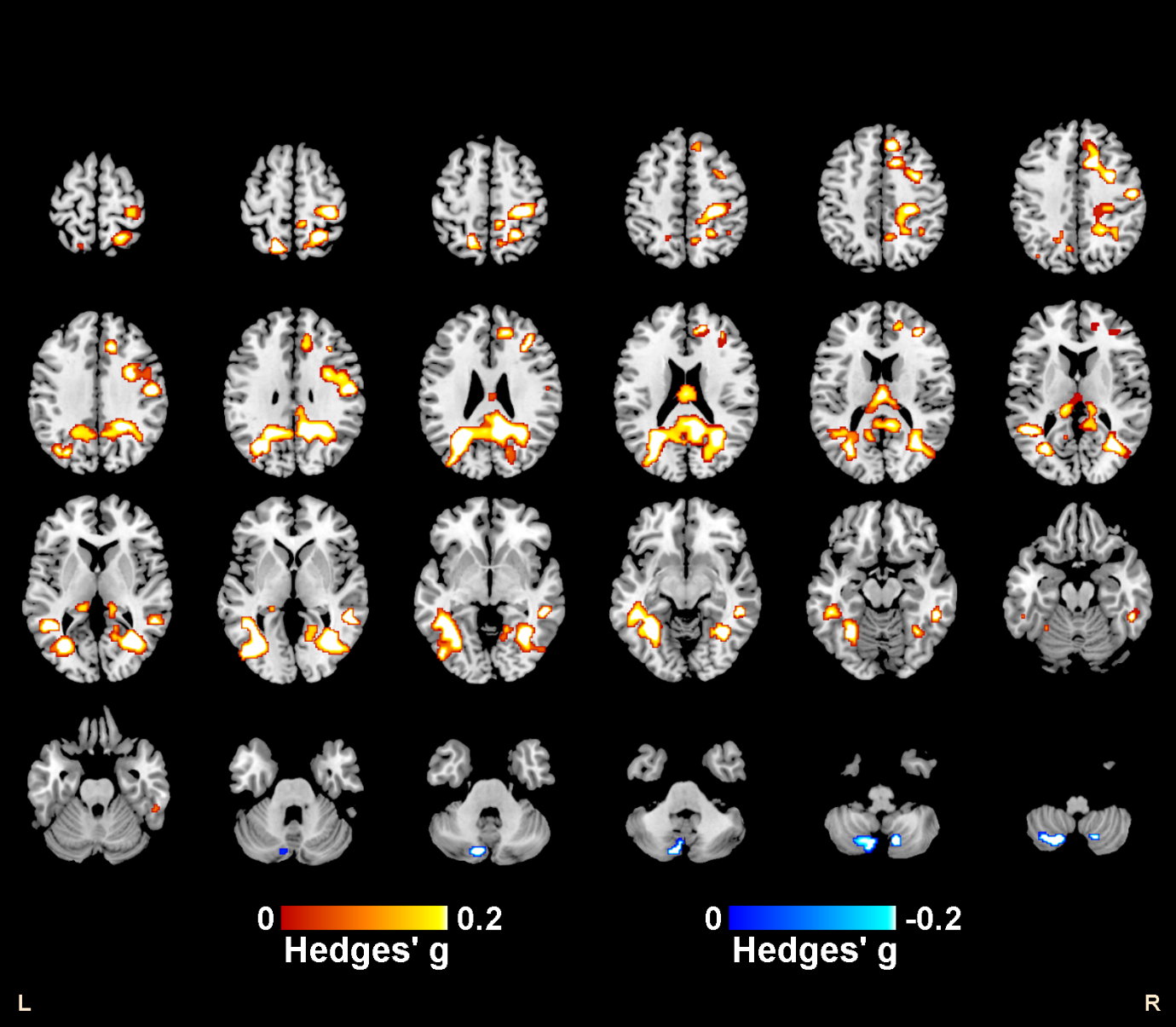


## Table S22 WM using Proportional scaling, controlling for age and ICV

| Peak region | MNI coordinate | Hedges' g | Z | Cluster size (voxels) | P value (FWE corrected) | I^2^ |
| --- | --- | --- | --- | --- | --- | --- |
| Right inferior network, inferior longitudinal fasciculus | 32,-64,2 | 0.33 | 4.822 | 8073 | 0.001 | 0.00 |
| Right superior longitudinal fasciculus III | 50,-14,34 | 0.28 | 4.029 | 1247 | 0.005 | 0.00 |
| Right arcuate network, posterior segment | 52,-44,0 | 0.27 | 3.889 | 294 | 0.010 | 0.00 |
| Corpus callosum | 36,34,24 | 0.26 | 3.735 | 122 | 0.018 | 0.00 |
| Left cerebellum, hemispheric lobule VIII | -20,-68,-50 | -0.36 | -5.242 | 255 | 0.001 | 0.00 |
| Right cerebellum, hemispheric lobule VIII | 14,-66,-48 | -0.38 | -5.535 | 49 | 0.007 | 0.00 |

Mean I^2^ = 6.79

## Figure S18 WM not controlling for any covariate


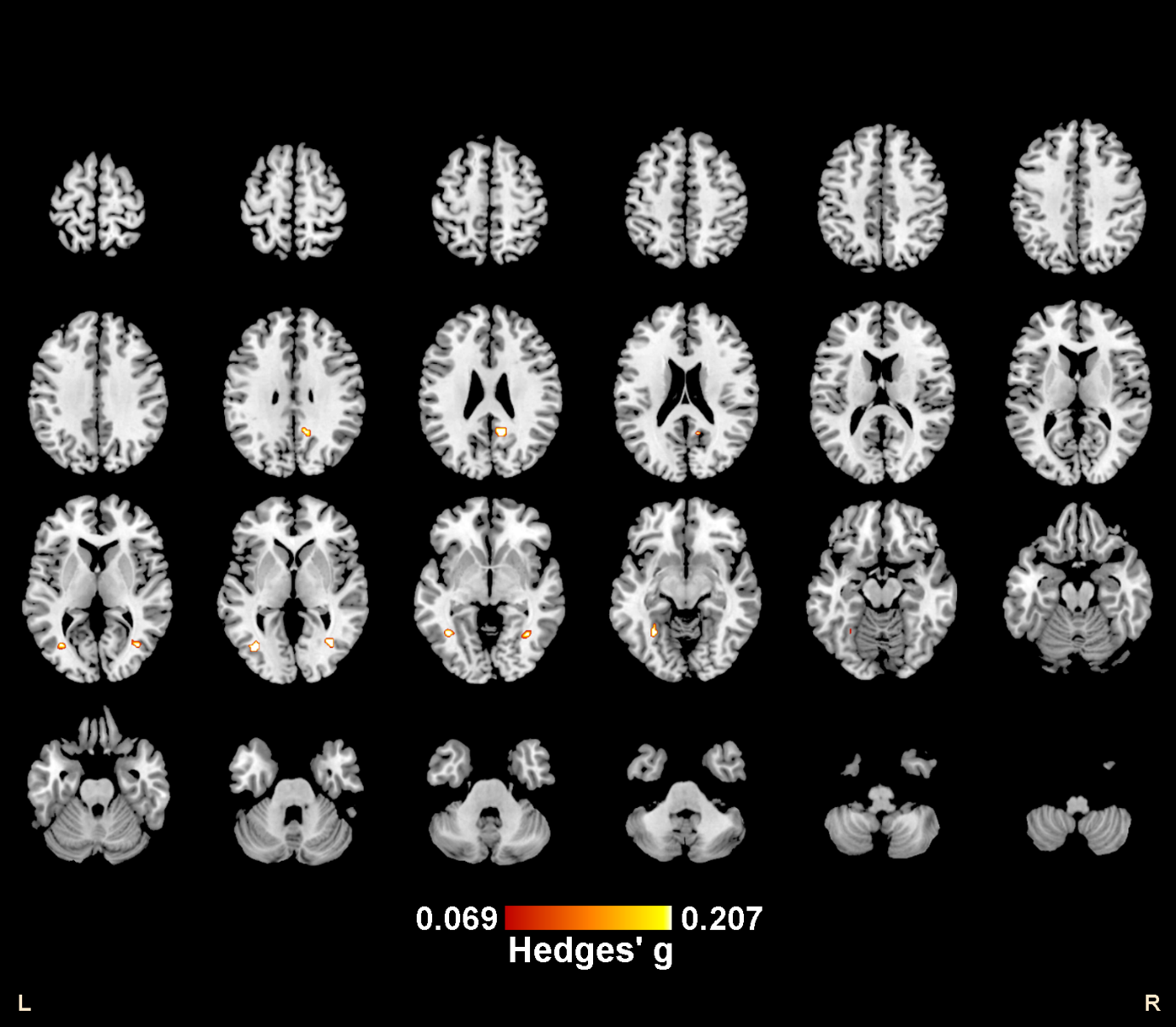


## Table S23 WM not controlling for any covariate

| Peak region | MNI coordinate | Hedges' g | Z | Cluster size (voxels) | P value (FWE corrected) | I^2^ |
| --- | --- | --- | --- | --- | --- | --- |
| Right inferior network, inferior longitudinal fasciculus | 34,-66,0 | 0.24 | 3.528 | 82 | 0.016 | 0.00 |
| Right median network, cingulum | 10,-54,24 | 0.24 | 3.521 | 77 | 0.015 | 0.00 |
| Left inferior network, inferior longitudinal fasciculus | -30,-56,-12 | 0.25 | 3.588 | 65 | 0.018 | 0.00 |

Mean I^2^ = 11.01

## Figure S19 GM volume analysis with no modulation in the VBM image processing and no covariates


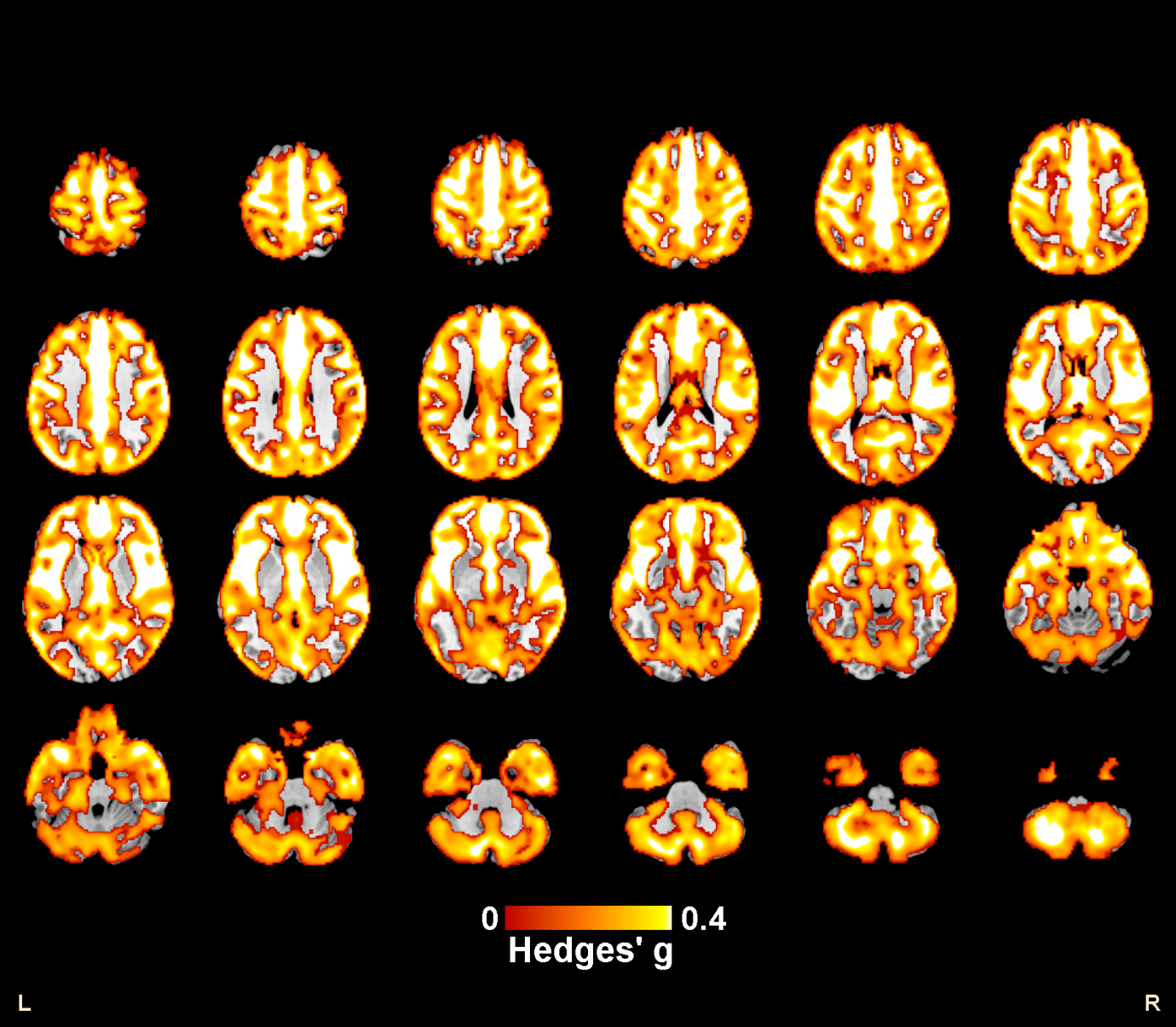


## Table S24 GM volume analysis with no modulation in the VBM image processing and no covariates

| Peak region | MNI coordinate | Hedges' g | Z | Cluster size (voxels) | P value (FWE corrected) | I^2^ |
| --- | --- | --- | --- | --- | --- | --- |
| **Right inferior frontal gyrus, opercular part, BA 48** | **44,14,26** | **0.57** | **8.273** | **155269** | **0.001** | **0.00** |
| Right middle temporal gyrus, BA 20 | 52,-12,-16 | 0.57 | 7.849 | subcluster | 0.001 | 6.03 |
| Right middle frontal gyrus, BA 9 | 30,40,34 | 0.54 | 7.805 | subcluster | 0.001 | 0.00 |
| Left insula, BA 47 | -32,22,-2 | 0.68 | 7.400 | subcluster | 0.001 | 36.18 |
| Left caudate nucleus, BA 25 | -2,6,-8 | 0.50 | 7.282 | subcluster | 0.001 | 0.00 |
| Right inferior frontal gyrus, triangular part, BA 45 | 52,36,16 | 0.50 | 7.176 | subcluster | 0.001 | 0.00 |

Mean I^2^ = 32.19

## Figure S20 WM volume analysis with no modulation in VBM image processing and no covariates


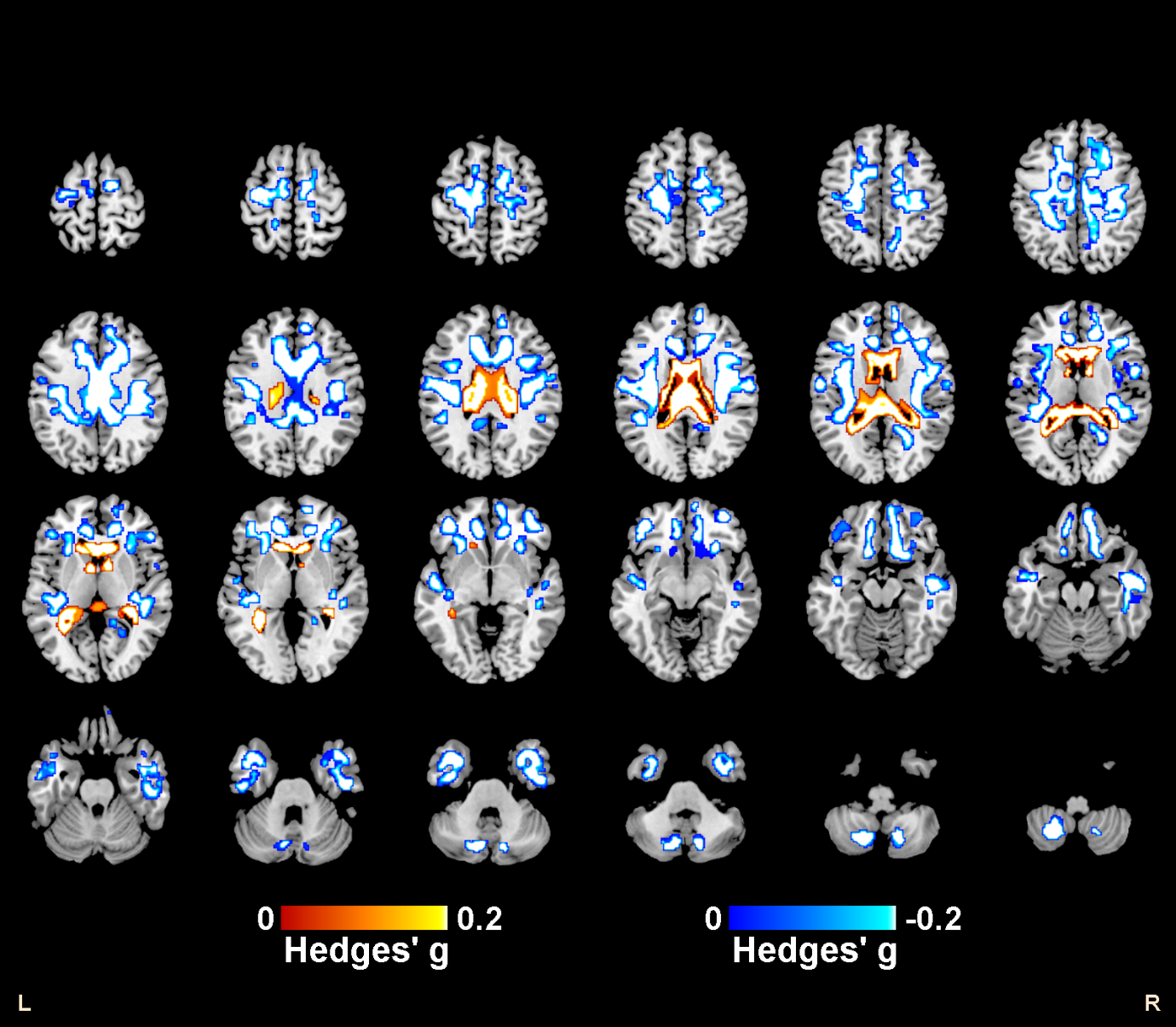


## Table S25 WM volume analysis with no modulation in VBM image processing and no covariates

| Peak region | MNI coordinate | Hedges' g | Z | Cluster size (voxels) | P value (FWE corrected) | I^2^ |
| --- | --- | --- | --- | --- | --- | --- |
| Corpus callosum | 0,20,6 | 0.39 | 5.627 | 4212 | 0.001 | 0.00 |
| Left median network, cingulum | -6,10,32 | -0.41 | -5.729 | 19075 | 0.001 | 6.19 |
| Left cerebellum, hemispheric lobule VIII | -18,-68,-48 | -0.47 | -6.863 | 424 | 0.001 | 0.00 |
| Right cerebellum, hemispheric lobule VIII | 12,-68,-44 | -0.49 | -7.033 | 152 | 0.004 | 0.00 |

Mean I^2^ = 9.41

## Figure S21 Regional GM volume difference between EOP and HC using different smooth kernels (2mm, 4mm, 8mm, 12mm)

With larger smoothing kernels, the results show more spatially extensive GM changes and the effect size also generally increases.
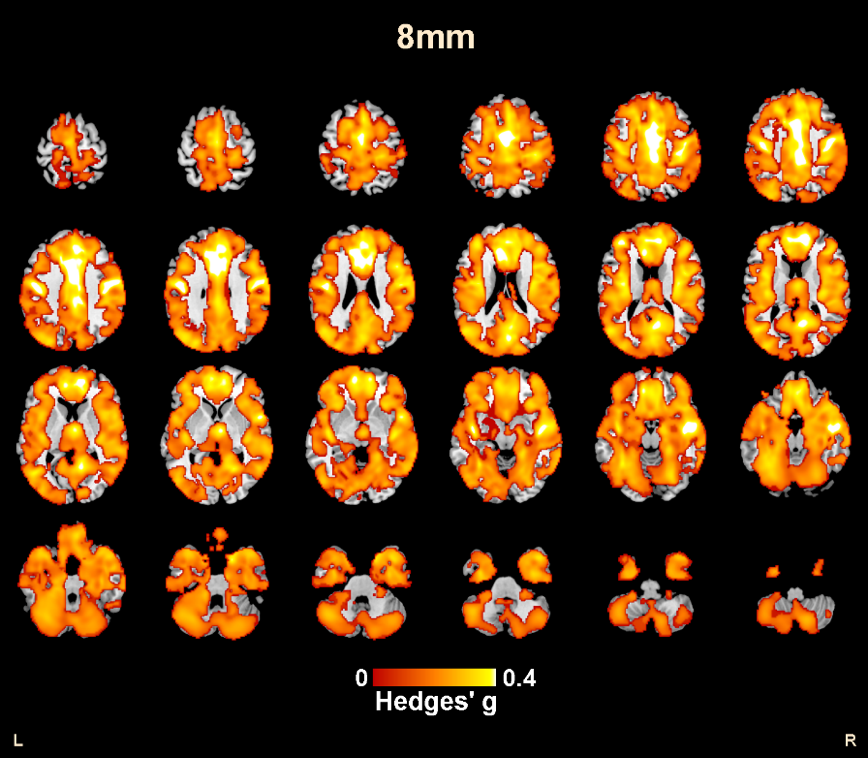

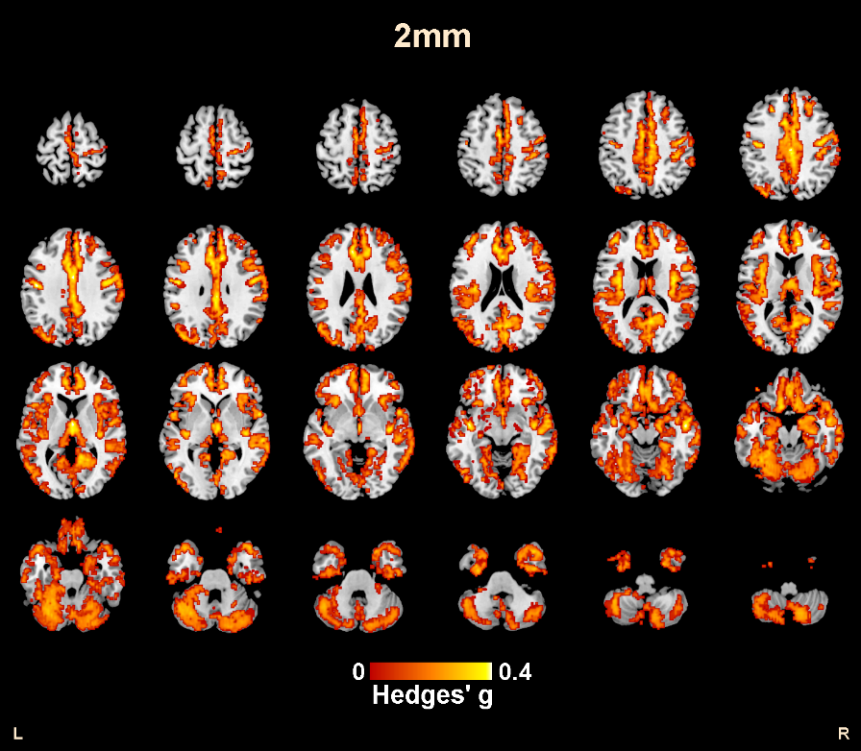

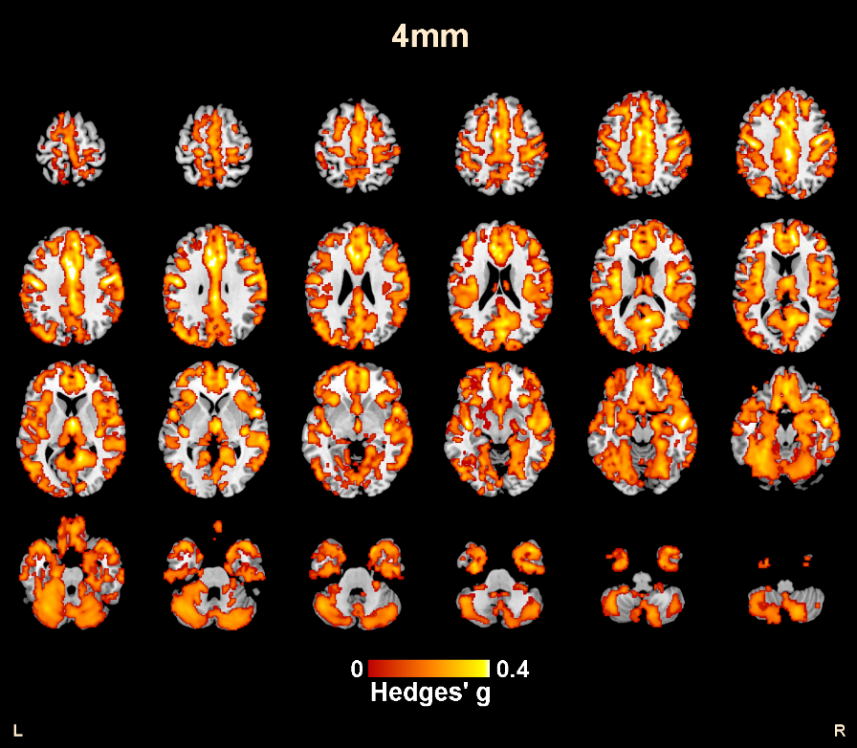

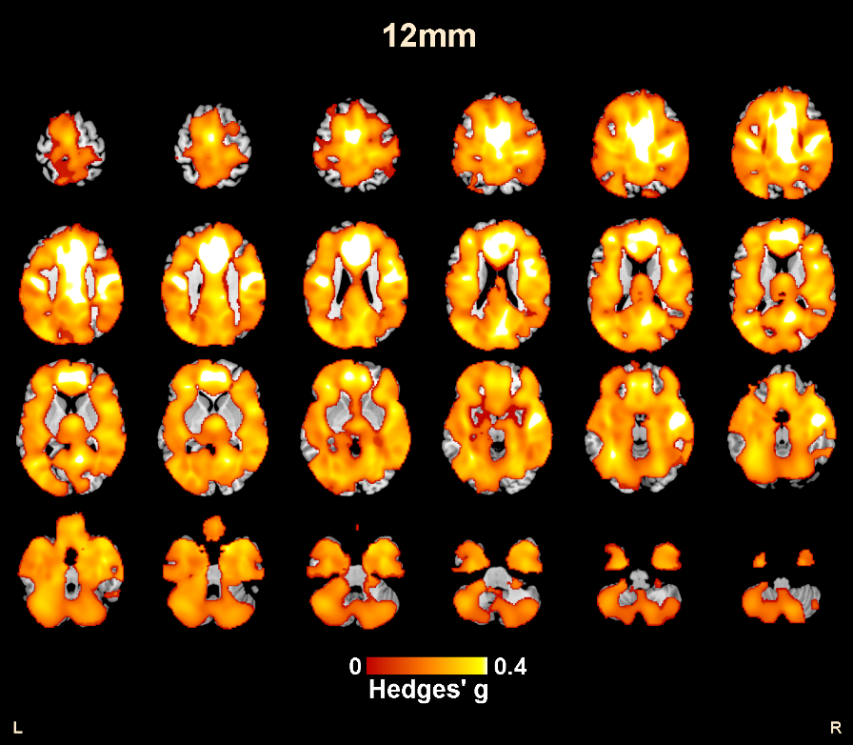


## Table S26 Regional GM volume difference between EOP and HC using different smooth kernels (2mm, 4mm, 8mm, 12mm)

| Smoothing kernel | Peak region | MNI coordinate | Hedges' g | Z | Cluster size (voxels) | P value (FWE corrected) | I^2^ |
| --- | --- | --- | --- | --- | --- | --- | --- |
| 2mm | Left median cingulate / paracingulate gyri | -4,-6,48 | 0.47 | 6.797 | 39169 | 0.001 | 0.00 |
|  | Left postcentral gyrus, BA4 | -48,-12,32 | 0.47 | 6.815 | 216 | 0.003 | 0.00 |
|  | Right postcentral gyrus, BA 3 | 42,-26,44 | 0.32 | 4.571 | 199 | 0.010 | 0.00 |
|  | Left middle frontal gyrus,BA9 | -22,26,44 | 0.41 | 5.601 | 20 | 0.015 | 1.69 |
|  | Right angular gyrus, BA 39 | 56,-58,28 | 0.29 | 4.241 | 19 | 0.019 | 0.00 |
| 4mm | Left median cingulate / paracingulate gyri | -4,-4,48 | 0.53 | 7.685 | 77555 | 0.001 | 0.00 |
| 8mm | Left median cingulate / paracingulate gyri | -4,-4,48 | 0.55 | 7.785 | 127295 | 0.001 | 0.00 |
| 12mm | Right median cingulate / paracingulate gyri | 6,-32,42 | 0.54 | 7.74 | 160693 | 0.001 | 0.00 |

Mean I^2^ (2mm) = 6.99, I^2^(4mm) = 8.62, I^2^(8mm) = 14.52, I^2^(12mm) = 19.73

## Figure S22 Regional WM volume difference between EOP and HC using different smooth kernels (2mm, 4mm, 8mm, 12mm)

No cluster passed thresholding when using 2mm smoothing kernel. With larger smoothing kernels, the results show spatially more extensive WM changes.
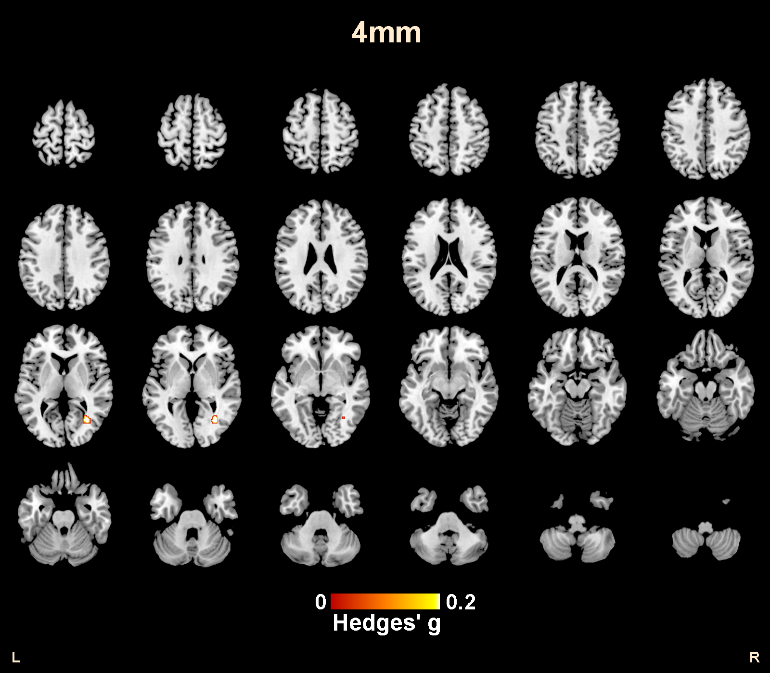

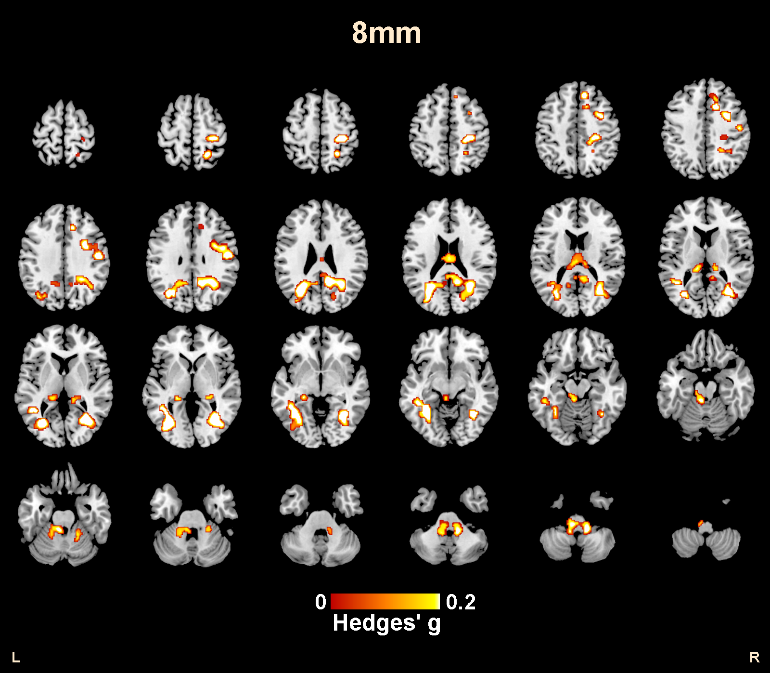

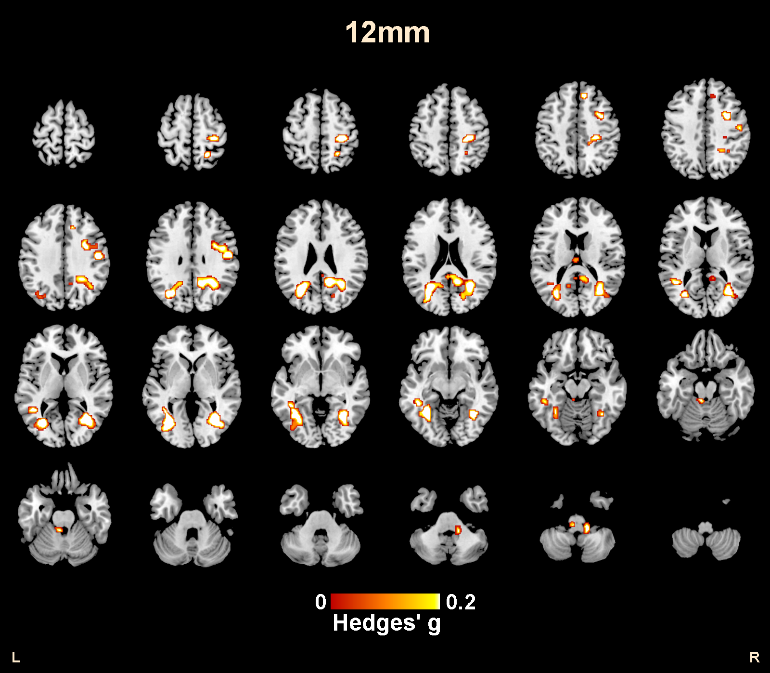


## Table S27 Regional WM volume difference between EOP and HC using different smooth kernels (2mm, 4mm, 8mm, 12mm)

| WM volume HC >EOP | Peak region | MNI coordinate | Hedges' g | Z | Cluster size (voxels) | P value (FWE corrected) | I^2^ |
| --- | --- | --- | --- | --- | --- | --- | --- |
| 2mm | no cluster passed thresholding |  |  |  |  |  |  |
| 4mm | Right inferior network, inferior longitudinal fasciculus | 32,-64,2 | 0.33 | 4.892 | 75 | 0.006 | 0.00 |
| 8mm | Right inferior network, inferior longitudinal fasciculus | 32,-64,2 | 0.33 | 4.839 | 2129 | 0.001 | 0.00 |
|  | Left inferior network, inferior longitudinal fasciculus | -34,-70,2 | 0.32 | 4.696 | 1924 | 0.001 | 0.00 |
|  | Right superior longitudinal fasciculus III | 50,-14,34 | 0.29 | 4.258 | 573 | 0.003 | 0.00 |
|  | Left anterior thalamic projections | -2,-20,16 | 0.22 | 3.204 | 437 | 0.017 | 0.00 |
|  | Left cerebellum | -8,-38,-22 | 0.22 | 3.219 | 319 | 0.017 | 0.00 |
|  | Corpus callosum | 10,32,46 | 0.24 | 3.431 | 121 | 0.017 | 0.00 |
|  | Right cerebellum | 22,-38,-28 | 0.20 | 2.899 | 60 | 0.023 | 0.00 |
| 12mm | Right inferior network, inferior longitudinal fasciculus | 32,-64,2 | 0.30 | 4.742 | 1844 | 0.001 | 0.00 |
|  | Left inferior network, inferior longitudinal fasciculus | -34, -70,2 | 0.31 | 4.534 | 1545 | 0.004 | 0.00 |
|  | Right superior longitudinal fasciculus III | 50,-14,34 | 0.29 | 4.285 | 466 | 0.004 | 0.00 |
|  | Middle cerebellar peduncles | 14,-38,-46 | 0.23 | 3.370 | 77 | 0.020 | 0.00 |
|  | Left cerebellum, hemispheric lobule III | -8,-38,-22 | 0.22 | 3.247 | 44 | 0.022 | 0.00 |
|  | Corpus callosum | 10,32,46 | 0.24 | 3.460 | 37 | 0.020 | 0.00 |
|  | Left anterior thalamic projections | -2,-20,16 | 0.21 | 3.064 | 24 | 0.024 | 0.00 |
|  | Left pons | 12,26,34 | 0.22 | 3.251 | 10 | 0.025 | 0.00 |

Mean I^2^(4mm) = 2.61, I^2^(8mm) = 5.52, I^2^(12mm) = 5.56

## Figure S23 Heterogeneity of effect size measured by I^2^ statistics (GM EOP vs. HC)

Mean heterogeneity across all regions measured by I^2^ is 14.52%.


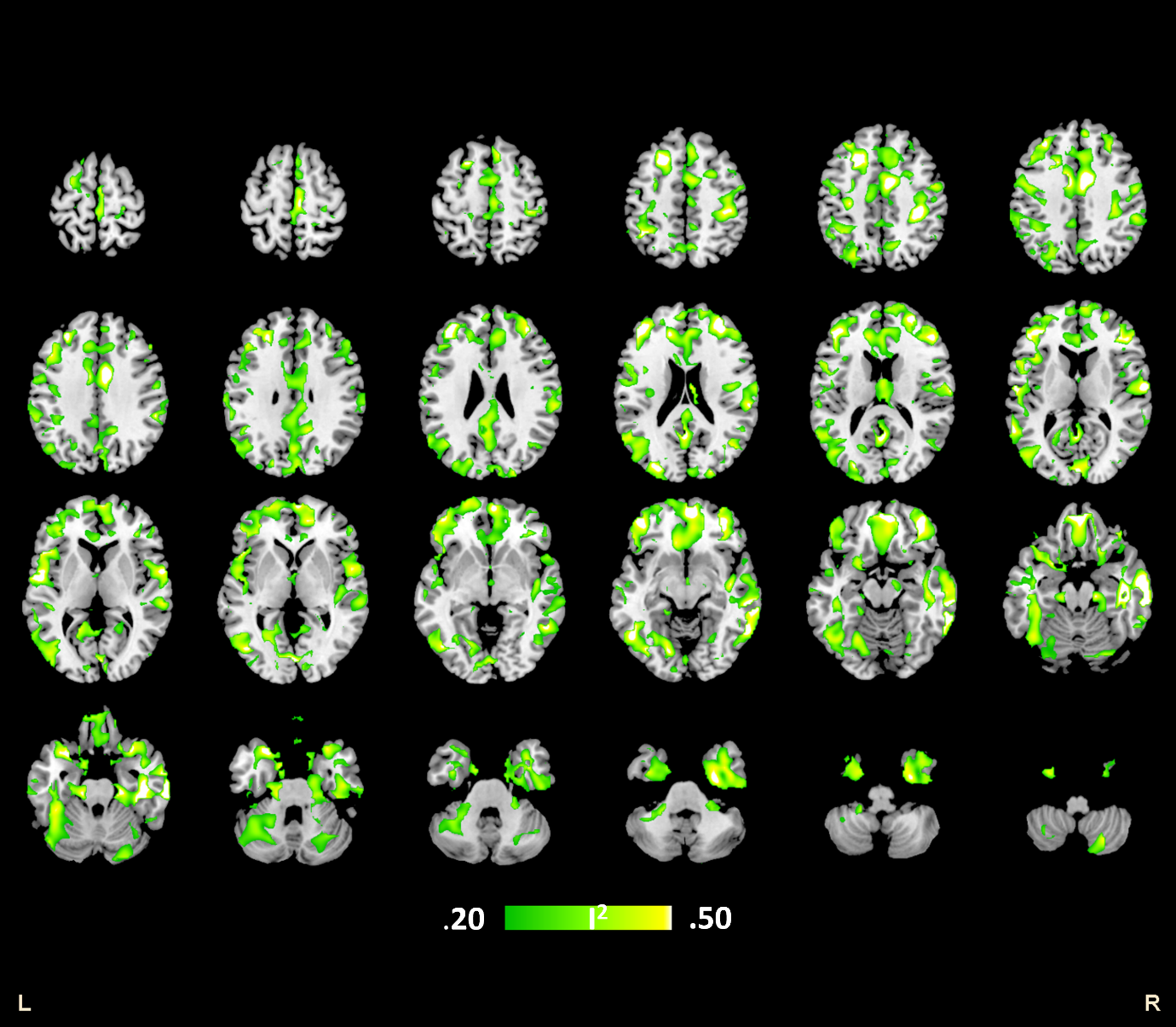


## Figure S24 Comparison of regional GM volume in EOP and Schizophrenia


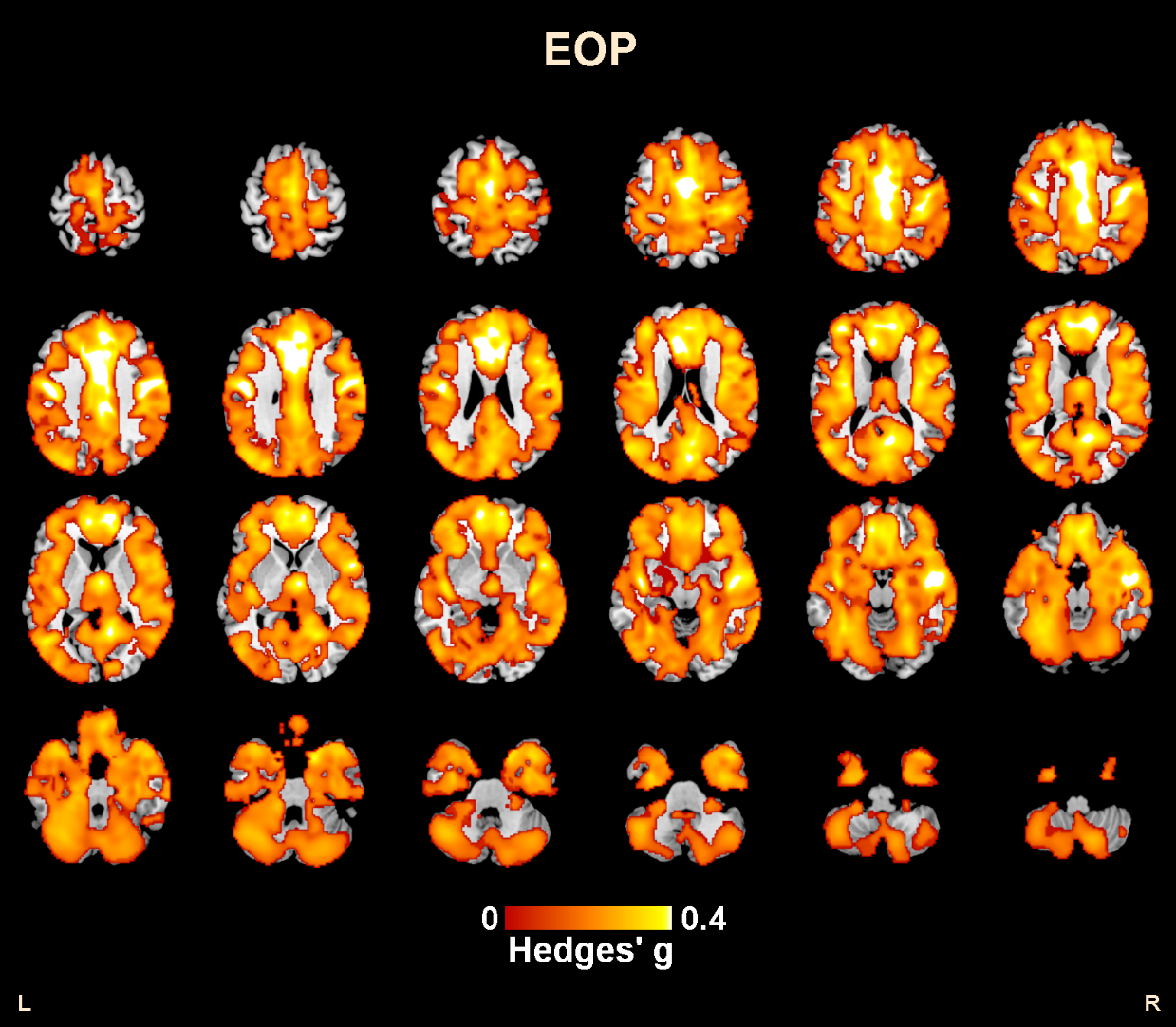

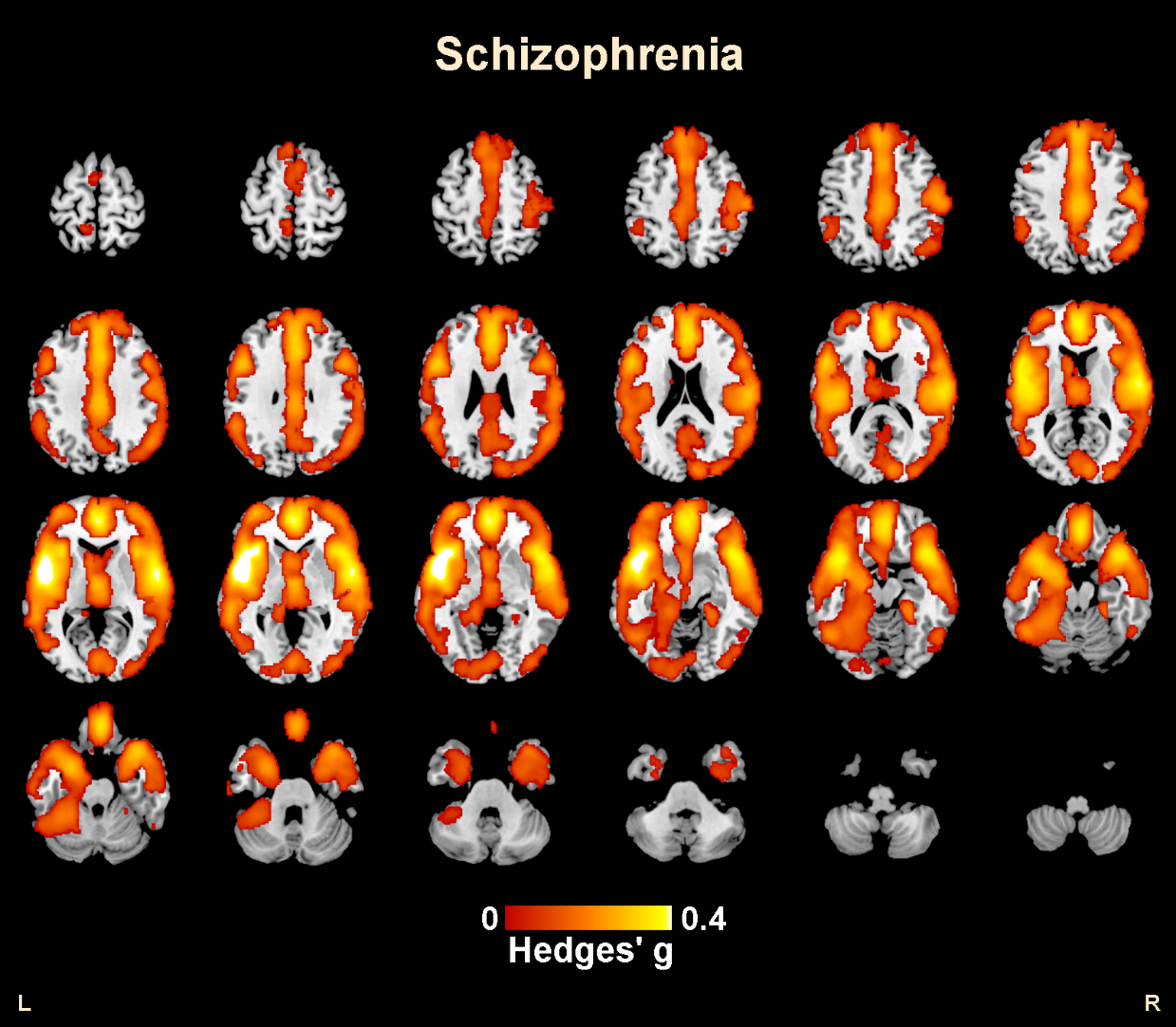

Supplement: Supplementary file 1 — Supplementary material A [file 41380_2023_2343_MOESM1_ESM.docx]
