# Supplementary material for: Mapping gray and white matter volume abnormalities in early-onset psychosis: an ENIGMA multicenter voxel-based morphometry study
Source: Mol Psychiatry. 2024 Jan 10;29(2):496–504. doi: 10.1038/s41380-023-02343-1 (PMC11116097; doi:10.1038/s41380-023-02343-1)
Supplement: Supplementary file 2 — Supplementary material B [file 41380_2023_2343_MOESM2_ESM.docx]

**Overview of how the ENIGMA VBM tool is used.**

The ENIGMA consortium consists of a large number of research sites who have collected T1 MRI from a group of patients and controls. Ideally for a given analysis, all raw data could be sent to a coordinating site, but this is not always possible because of concerns regarding data privacy. For this reason, the ENIGMA VBM tool (<https://sites.google.com/view/enigmavbm>) has been developed so that sites can choose between transferring individual data or aggregate group data. In the current study each site processed their data using the tool and transferred the processed group data to the coordinating site for subsequent meta-analysis using Seed-based d-Mapping (SDM).

**Technical Description**

The ENIGMA VBM tool is written in MATLAB and calls various procedures from SPM12. The software is backward compatible to MATLAB 2007 to ensure maximum usability. The version of the tool described below and used in the current study was ENIGMA VBM tool version 1.013. In addition to the description below, Figure A shows the inputs and outputs of the script and a summary of the processing steps.

Data input

The user is first asked to enter information which will help the coordinating site identify the data (PI name, email and city). The software requests the CovariatesVBM.csv file which is a comma-separated values (CSV) spreadsheet. This is a standardized file that was initially developed when ENIGMA first started analysis using FreeSurfer and we have retained the format for ease of use. The tool requires the 1st column in the CSV file to be the subject ID, followed by diagnosis, age and sex. Any number of additional columns can be included for encoding clinical data. The user is then asked to select the T1 MRI data for patients and then controls and to check the orientation of a random sample of data. The user is asked to select the ‘CNR.nii’ file supplied with the tool which contains regions of interest (ROIs) in MNI space to sample gray matter (GM), white matter (WM), cerebrospinal fluid (CSF), air and bone. This is used to calculate contrast-to-noise ratio (CNR) statistics of the site’s data which may be used by the coordinating site to assess image quality.

Data Processing

The core part of the script is a standard DARTEL VBM process. This has been previously described ^1^ and is also summarised below. For all steps, default SPM settings have been used unless otherwise stated. Firstly, for the **Segment** step, individual T1 MRI data is segmented, producing gray and white matter ‘native’ and ‘DARTEL imported’ images. The DARTEL imported images are processed by the **DARTEL create template** step which iteratively aligns GM and WM segmented images by adjusting three parameters for each voxel to generate its own average template. These parameters are output as flow fields for each subject which encode the movement for each voxel from the imported image to match the template. The next step is the **Normalise to MNI** procedure**.** This step applies the flow field to the corresponding native segmentation for each subject, and the resulting segmented image is normalised to MNI space. This step provides the option to apply modulation, which ensures that each voxel represents true volume, as the procedure takes into account the amount each voxel has been dilated or compressed during normalisation. The ENIGMA VBM tool applies modulation in the standard analysis but also applies no modulation for the sensitivity analysis. The image is smoothed by a gaussian kernel with FWHM (full width half maximum) of 8mm (adjusted from 2 to 12mm in the sensitivity analysis). The final result is smoothed segmented images in MNI space.

Statistical Analysis

In the standard statistical analysis, the smoothed segmented images are compared between patients and controls, covarying for age and ICV. The ENIGMA VBM tool uses SPM basic models for the design specification, default classical model estimation (which uses restricted maximum likelihood), and the SPM contrast manager in batch mode. A sensitivity analysis is conducted to examine how robust the case-control differences are to variations in the covariates selected, including, age, ICV, sex, total GM and total WM. Additional sensitivity analyses test different parameters in image processing in SPM, including using proportional scaling (scaling the brain volume of each voxel by the fraction of ICV rather than using ICV as a covariate), no modulation and smoothing kernels from 2-12mm. In the patient group, regression analyses are conducted for each clinical variable in the CovariatesVBM.csv (e.g., examining where in the brain GM decreases with symptom severity). For each regression, 2 versions are produced: one covarying for ICV and age, and one covarying for ICV, age, and sex. In the current study the regression controlling for ICV, age and sex was used because of concerns of sex differences in clinical variables such as age of onset.

Quality Control

A number of quality control steps are integrated into the VBM tool. The user is asked to check the orientation of the MRI images, and 2D screenshots are also saved at each stage along the VBM pipeline which is visually assessed at the coordinating site. Voxel sizes, age and gender in each group, and CNR and templates are inspected at the coordinating site. To ensure that the demographic and clinical data is correctly matched with the MRI data, the tool checks that the mean male ICV is larger than mean female ICV. This finding has a large effect size of Cohen's d=1.3 and would be expected in all large adult MRI datasets. However, as the effect is less robust in adolescents, this requirement was relaxed in the current study.

Tool Validation

To validate the tool an analysis with 36 subjects from the IXI dataset (<https://brain-development.org/ixi-dataset/>) was undertaken manually. 36 IXI subjects were randomly divided into two groups as ‘patients’ and ‘controls. All processing was done manually in VBM SPM12 as well as using ENIGMA VBM tool. Outputs from each process, i.e., rc1.nii, u_rc1.nii, were compared between manual process and VBM tool using Pearson’s correlation. The summary of the demographics produced by the tool exactly matched the values calculated manually, and the Pearson’s correlation between the manual and the automated process for all image outputs was R ≥ 0.999.


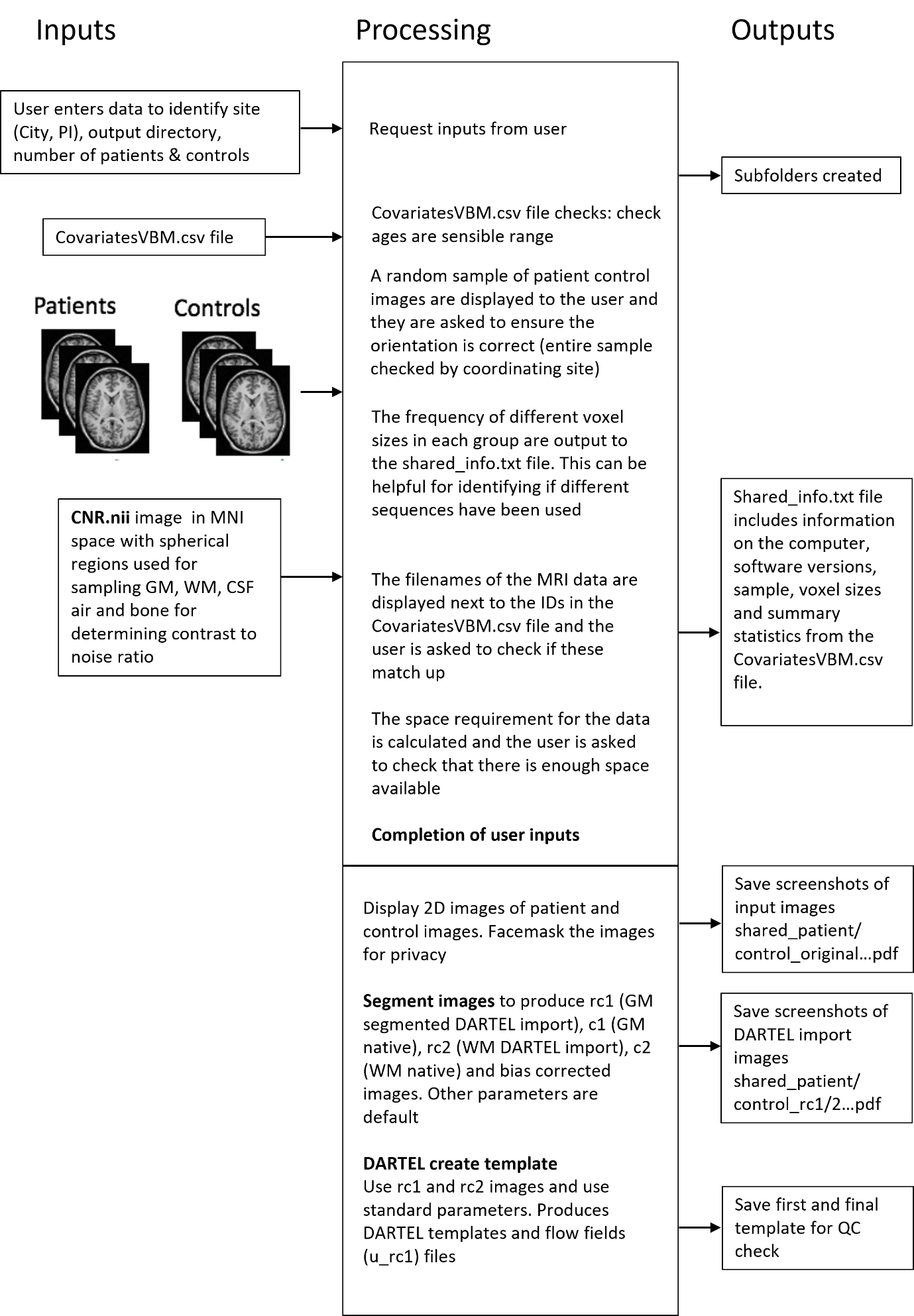


**Figure A:** Diagram showing how the ENIGMA VBM tool functions


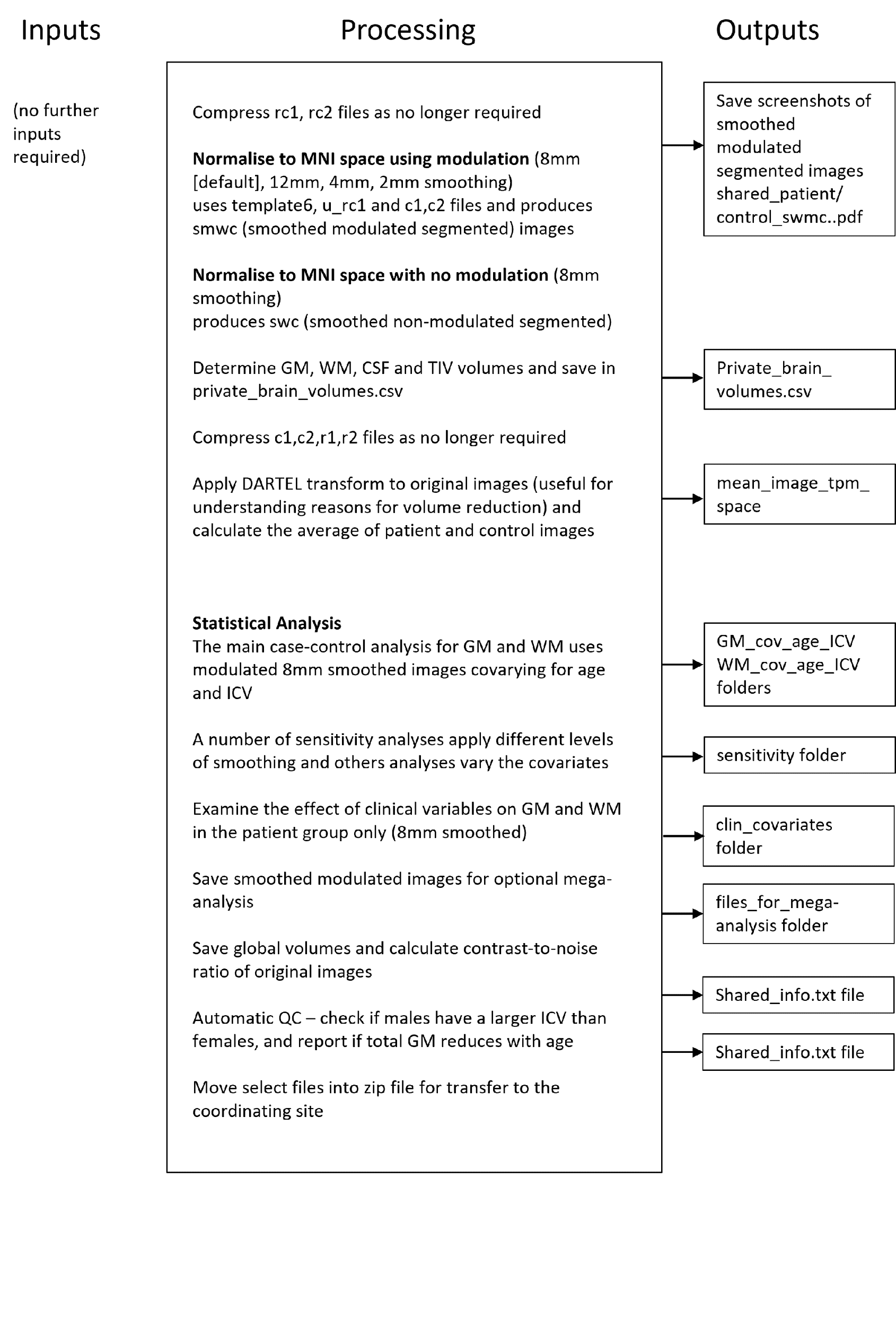


**Figure A, continued:** Diagram showing how the ENIGMA VBM tool functions

**SDM analysis**

As described in the main manuscript, SDM-PSI version 6.21 was used to meta-analyse the T-maps produced from each cohort. We used the Preprocessing, Mean, FWE correction and Threshold steps. Standard settings were used for all procedures, except the selection of the mask in the Preprocessing step. Instead of using the gray/white matter mask, we chose the whole brain mask to ensure all brain regions were covered. This is because we noticed that some parts of the globus pallidus was not included in the analysis.

**References**

1. Ashburner J. VBM tutorial. *Tech repWellcome Trust Centre for Neuroimaging, London, UK* 2010.
